# Association of the Hospital Readmissions Reduction Program With Mortality During and After Hospitalization for Acute Myocardial Infarction, Heart Failure, and Pneumonia

**DOI:** 10.1001/jamanetworkopen.2018.2777

**Published:** 2018-09-28

**Authors:** Rohan Khera, Kumar Dharmarajan, Yongfei Wang, Zhenqiu Lin, Susannah M. Bernheim, Yun Wang, Sharon-Lise T. Normand, Harlan M. Krumholz

**Affiliations:** 1 Division of Cardiology, University of Texas Southwestern Medical Center, Dallas; 2 Clover Health, Jersey City, New Jersey; 3 Center for Outcomes Research and Evaluation, Yale-New Haven Hospital, New Haven, Connecticut; 4 Section of Cardiovascular Medicine, Department of Internal Medicine, Yale School of Medicine, New Haven, Connecticut; 5 Section of General Internal Medicine, Department of Internal Medicine, Yale School of Medicine, New Haven, Connecticut; 6 Department of Biostatistics, T.H. Chan School of Public Health, Harvard University, Boston, Massachusetts; 7 Department of Health Care Policy, Harvard Medical School, Boston, Massachusetts; 8 Department of Health Policy and Management, Yale School of Public Health, New Haven, Connecticut

## Abstract

**Importance:**

The US Hospital Readmissions Reduction Program (HRRP) was associated with reduced readmissions among Medicare beneficiaries hospitalized for acute myocardial infarction (AMI), heart failure (HF), and pneumonia. It is important to assess whether there has been a signal for concomitant harm with an increase in mortality.

**Objective:**

To evaluate whether the announcement or the implementation of HRRP was associated with an increase in either in-hospital or 30-day postdischarge mortality following hospitalization for AMI, HF, or pneumonia.

**Design, Setting, and Participants:**

In this cohort study, using Medicare data, all hospitalizations for AMI, HF, and pneumonia were identified among fee-for-service Medicare beneficiaries aged 65 years and older from January 1, 2006, to December 31, 2014. These were assessed for changes in trends for risk-adjusted rates of in-hospital and 30-day postdischarge mortality after announcement and implementation of the HRRP using an interrupted time series framework. Analyses were done in November 2017 and December 2017.

**Exposures:**

Announcement of the HRRP in March 2010, and implementation of its penalties in October 2012.

**Main Outcomes and Measures:**

Monthly risk-adjusted rates of in-hospital and 30-day postdischarge mortality.

**Results:**

The sample included 1.7 million AMI, 4 million HF, and 3.5 million pneumonia hospitalizations. Between 2006 and 2014, in-hospital mortality decreased for the 3 conditions (AMI, from 10.4% to 9.7%; HF, from 4.3% to 3.5%; pneumonia, from 5.3% to 4.0%) while 30-day postdischarge mortality decreased from 7.4% to 7.0% for AMI (*P* for trend < .001), but increased from 7.4% to 9.2% for HF (*P* for trend < .001) and from 7.6% to 8.6% for pneumonia (*P* for trend < .001). Before the HRRP announcement, monthly postdischarge mortality was stable for AMI (slope for monthly change, 0.002%; 95% CI, −0.001% to 0.006% per month), and increased by 0.004% (95% CI, 0.000% to 0.007%) per month for HF and by 0.005% (95% CI, 0.002% to 0.008%) per month for pneumonia. There were no inflections in slope around HRRP announcement or implementation (*P* > .05 for all). In contrast, there were significant negative deflections in slopes for readmission rates at HRRP announcement for all conditions.

**Conclusions and Relevance:**

Among Medicare beneficiaries, there was no evidence for an increase in in-hospital or postdischarge mortality associated with HRRP announcement or implementation—a period with substantial reductions in readmissions. The improvement in readmission was therefore not associated with any increase in in-hospital or 30-day postdischarge mortality.

## Key Points

**Question:**

Was the announcement or implementation of the Hospital Readmissions Reduction Program (HRRP) associated with an increase in mortality following hospitalization for acute myocardial infarction, heart failure, or pneumonia among Medicare beneficiaries?

**Findings:**

In this cohort study, between 2006 and 2014, in-hospital mortality decreased for the 3 conditions while 30-day postdischarge mortality decreased for acute myocardial infarction but increased for heart failure and pneumonia. Before the announcement of the HRRP, postdischarge mortality was stable for acute myocardial infarction and increasing for heart failure and pneumonia, and there were no inflections in slope around the announcement or implementation of the HRRP.

**Meaning:**

There was no evidence for increase in in-hospital or postdischarge mortality associated with the HRRP announcement or implementation—a period with substantial reductions in readmissions.

## Teaser

This cohort study uses Medicare data to evaluate whether the announcement or implementation of the Hospital Readmissions Reduction Program (HRRP) was associated with an increase in either in-hospital or 30-day postdischarge mortality following hospitalization for acute myocardial infarction (AMI), heart failure (HF), or pneumonia.

## Introduction

The Hospital Readmissions Reduction Program (HRRP) was introduced in 2010 with the goal of reducing early readmissions following hospitalization for common medical conditions.^1^ Acute myocardial infarction (AMI), heart failure (HF), and pneumonia were the 3 conditions included in the program at its inception, and the HRRP sought to improve health system performance and reduce readmissions for these conditions by creating financial incentives for hospitals to invest in improved clinical care of patients during and after hospitalization.^1,2,3^ The HRRP implemented financial penalties at hospitals with rates of 30-day readmission for these 3 conditions in excess of the national average. The introduction of the program was associated with a substantial reduction in readmissions nationally.^2,4^

The success of the HRRP with reducing readmission mandates that we ensure that no harm has accrued. A balancing measure to readmission is mortality, and harm with reduced readmissions under the HRRP may manifest as an increase in mortality with the institution of the program. Mortality is a concern because physicians may have a financial incentive to reduce hospitalizations, and may inadvertently refuse required hospitalizations.^5^ The financial penalties in the program may also inadvertently worsen the quality of care at hospitals with limited resources. While hospitals that reduced readmissions, on average, also had reductions in mortality,^6^ there have been concerns that the HRRP may have led to changes in the ecological trends for mortality for the 3 conditions. Specifically, hospitals may have adopted practices that resulted in higher mortality, even if these measures were unsuccessful in reducing readmissions.^7,8^ Therefore, a comprehensive review of the relationship of the HRRP announcement and implementation with time trends in mortality during hospitalization and within a 30-day period after discharge for the 3 target conditions is essential to critically evaluate these concerns.

We therefore used Medicare standard analytic files, which include the entire fee-for-service Medicare population, to assess mortality during hospitalization and within 30 days of discharge following a hospitalization for 1 of the 3 HRRP conditions between the years of 2006 and 2014. Specifically, we examined whether time trends for in-hospital and postdischarge mortality in this population changed at the initial announcement of the HRRP or at the implementation of the penalty phase.

## Methods

### Study Population

We identified all inpatient hospitalizations for AMI, HF, and pneumonia with a discharge date between January 1, 2006, and December 31, 2014, among fee-for-service Medicare beneficiaries aged 65 years and older at any nonfederal acute care hospital in the United States. Hospitalizations in which the patients did not have a full year of preadmission enrollment in fee-for-service Medicare, were transferred from another acute hospital (for in-hospital mortality outcome), were transferred to another acute care hospital (for 30-day postdischarge mortality and readmission outcomes), or left the hospital against medical advice were excluded. In assessments of postdischarge mortality and readmission, patients alive but without at least 30 days of postdischarge follow-up were also excluded, and hospitalizations for the same condition within 30 days of an index hospitalization were not considered index events. The approach used to construct the study population is the same as that used by the Centers for Medicare & Medicaid Services (CMS) in assessing hospital mortality and readmission performance.^4^ The patient selection approach is outlined in the eMethods and eFigures 1 through 6 in the Supplement. Institutional review board approval, including waiver of the requirement of participant informed consent, was provided by the Yale University Human Investigation Committee as the data were deidentified. The study was reported in accordance with the Strengthening the Reporting of Observational Studies in Epidemiology (STROBE) reporting guideline.

### Data Sources

We used the Medicare standard analysis data files (2006 to 2014) and defined AMI, HF, and pneumonia hospitalizations as any hospitalization with a primary discharge diagnosis of the respective conditions, as in prior studies.^2,4,6^ We also obtained information on patient characteristics including demographics (age and sex) and mortality using the denominator files, and relevant comorbid conditions from the diagnosis codes in the insurance claims submitted to Medicare across inpatient and outpatient care settings.^9,10,11,12,13,14,15,16^

### Study Outcomes

Our coprimary study outcomes were in-hospital and 30-day postdischarge all-cause mortality. These were defined as death from any cause during the hospitalization and within 30 days of discharge, respectively. We also assessed 30-day all-cause unplanned readmission, ie, hospitalization owing to any cause within 30 days of discharge, excluding planned readmissions using the CMS algorithm.^17^ The study outcomes and their definitions are identical to those in prior studies.^4,6^ We assessed 2 secondary mortality outcomes: (1) either in-hospital or 30-day postdischarge mortality, representing death during hospitalization or within 30-day of discharge, as a recommended outcome in a recent report by the Medicare Payment Advisory Commission that encompasses the entirety of hospitalization-related mortality without being sensitive to changes in length of stay over time,^18^ and (2) 30-day postadmission mortality, which is a publicly reported measure of death within 30 days of hospitalization.

### Statistical Analysis

Our approach involved first adjusting for patient risk and comorbidity for each patient discharge, and creating a monthly risk-adjusted estimate of each of the study outcomes. Next, using the monthly risk-adjusted estimates, we assessed temporal trends based on an interrupted time series model. Finally, we examined trends in the monthly risk-adjusted estimates using a nonparametric approach.

#### Adjusted Monthly Hospital Mortality and Readmission Rates

We calculated monthly admission-level risk-adjusted rates of 5 outcomes for each of the 108 calendar months using the discharge date during our study period (January 1, 2006, through December 31, 2014) as the most recent year with data coded using the *International Classification of Diseases, Ninth Revision*.^19^ To account for changes in patient characteristics over time as well as clustering of patients within hospitals, we constructed hierarchical logistic regression models with each of the outcomes separately as dependent variables, patient characteristics (age, sex, and comorbid conditions) as independent variables, and a random intercept for the hospital.

The comorbid conditions included in the model are the same as those that have been used in prior studies^6^ and in CMS quality measures.^10,12,13,20,21,22^ The risk-adjusted rate of each outcome for hospitalizations in each month during this period were obtained using indirect standardization, defined as the ratio of the observed rate of the outcome to the expected rate of the respective outcome in the risk-adjustment model above, multiplied by the unadjusted rate of the outcome observed in the 9-year study period. Further, to examine for changes in risk-factor burden over time, the linear combination of covariates from the model for postdischarge mortality in 2006 were used to calculate an estimate of severity of an average patient each year, and then reported as a mortality risk score indexed to the first year of the study.

#### Trends in Hospital Mortality and Readmission Rates

We used an interrupted time series framework to assess the relationship between monthly risk-adjusted rates of our outcomes, and the 2 relevant time points in the implementation of the HRRP^7^: April 2010, the month following the announcement of the HRRP, and October 2012, the month when financial penalties under the program were initiated. We parameterized month as a count from 1 to 108, assumed the adjusted estimates were linearly associated within a month, and assumed that after accounting for month, no serial correlation existed. We weighted each hospital monthly risk-adjusted estimate by the inverse of its variance. We parameterized the HRRP effect to have a gradual relationship with the mortality (or readmission) rates, thereby only affecting the slope of the mortality (or readmission) regression curve. In this analysis, we constructed a linear spline across monthly risk-adjusted rates of each outcome with knots at calendar months corresponding to the HRRP’s announcement (April 2010), and the implementation of its financial penalties (October 2012). We assessed the slopes of the spline both before and after each transition, and for changes in the slope of the time trends in these outcomes at these predefined knots.

We fit a Loess model, a nonparametric regression approach based on the use of a locally weighted polynomial regression and a variable span least squares smoothing procedure, to estimate the temporal relationship between months and observed as well as rates of risk-adjusted outcomes across the study period.^23,24^

The level of significance was set at .05. To maintain a family-wise type I error rate of 0.05, we used the Holm-Bonferroni method of multiple comparison testing for our coprimary outcomes for each of the 3 conditions.^25^ This defined a test-specific significance level of .05/(number of tests in analysis + 1 − rank of test *P* value from lowest to highest). Since our study focused on assessing harms, the lower type II error rate in the Holm-Bonferroni approach makes it more appropriate than the traditional Bonferroni correction.^25^

Analyses were performed in November and December of 2017 and May and June of 2018 by Dr Khera and Mr Yongfei Wang using SAS, version 9.4 (SAS Institute Inc), Stata, version 14 (StataCorp), and R, version 3.4.3 (The R Foundation).

## Results

**Figure 1.**
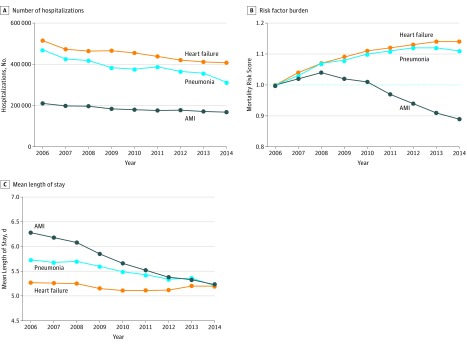
Calendar-Year Trends in Number of Hospitalizations, Risk Factor Burden, and Length of Stay A, Calendar-year trends in the number of hospitalizations for acute myocardial infarction (AMI), heart failure, and pneumonia. B, Cumulative burden of risk factors represented by the mean of the linear predictors (patient-level covariates combined with their corresponding regression coefficients from the risk-adjustment models for post–30-day mortality based on the 2006 data), with 2006 as the reference year. The dotted line indicates the reference level of risk score. C, Mean length of stay for hospitalizations.

We identified 1.7 million hospitalizations for AMI, 4 million for HF, and 3.5 million for pneumonia among fee-for-service Medicare beneficiaries from January 1, 2006, to December 31, 2014. The characteristics of patients, overall, and for each calendar year in the study, are presented in eTables 1 to 3 in the Supplement. Notably, the number of hospitalizations for each of the 3 HRRP conditions decreased during this period (Figure 1A). The risk factor burden, which was assessed as a summary mortality risk score for an average patient each year, decreased for AMI hospitalizations, but increased for HF and pneumonia hospitalizations (Figure 1B). The average length of stay decreased for AMI and pneumonia, but remained substantively unchanged for HF (Figure 1C).

Between 2006 and 2014, unadjusted in-hospital mortality rates decreased from 10.4% to 9.7% for AMI, from 4.3% to 3.5% for HF, and from 5.3% to 4.0% for pneumonia. Over the same period, unadjusted 30-day postdischarge mortality rates decreased from 7.4% to 7.0% for AMI, but increased from 7.4% to 9.2% for HF and from 7.6% to 8.6% for pneumonia. Unadjusted 30-day readmission rates decreased from 18.9% to 16.0% for AMI, 23.0% to 21.4% for HF, and 17.4% to 16.5% for pneumonia (*P* < .001 for all) (eTables 1 through 3 and eFigures 1 through 3 in the Supplement). For the outcome of in-hospital or 30-day postdischarge mortality, unadjusted mortality decreased from 17.9% in 2006 to 15.0% in 2014 for AMI, increased from 12.7% in 2006 to 13.6% in 2014 for HF, and remained unchanged at approximately 13.2% for pneumonia. There was a similar trend for unadjusted 30-day postadmission mortality, which decreased for AMI, but increased for HF.

### Trends in Risk-Adjusted Mortality and Readmissions

#### Acute Myocardial Infarction

**Figure 2.**
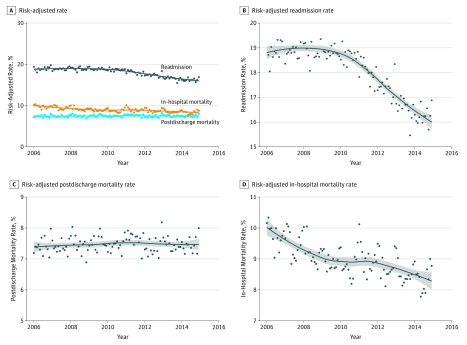
Time Trends in Risk-Adjusted Mortality and Readmissions for Acute Myocardial Infarction (AMI) A, Trend plot for risk-adjusted monthly in-hospital mortality, postdischarge 30-day mortality, and 30-day readmission. Trend lines represent nonparametric locally weighted regression (Loess) lines. B, C, and D, Trend lines with expanded axes. The gray band represents the 95% confidence interval for trend lines.

Between 2006 and 2014, rates of both risk-adjusted in-hospital mortality and readmission for AMI decreased, while postdischarge mortality remained unchanged (slope for monthly change, 0.002%; 95% CI, −0.001% to 0.006% per month) (Figure 2). Monthly risk-adjusted in-hospital mortality rates decreased by 0.021% (95% CI, −0.027% to −0.015%) before the announcement of the HRRP, with no decrease during the period after the HRRP announcement and before its penalties were implemented (−0.001% per month; 95% CI, −0.010% to 0.009% per month). In-hospital mortality continued to decline by 0.021% (95% CI, −0.036% to −0.005%) per month in the HRRP penalty period (Table). In contrast, there were no significant changes in risk-adjusted 30-day postdischarge mortality rates in any of the 3 periods relevant to the HRRP, without any inflections corresponding to either its announcement or its penalties (*P* > .05 for both). Further, while there were no significant changes in risk-adjusted 30-day readmission rates in the pre-HRRP period, readmission rates decreased significantly during both the post-HRRP announcement (−0.059% per month; 95% CI, −0.069% to −0.050% per month) and HRRP penalty periods (−0.042% per month; 95% CI, −0.057% to −0.027% per month) (Table).

**Table.** Interrupted Time Series for Risk-Adjusted In-Hospital Mortality, and 30-Day Risk-Adjusted Postdischarge Mortality and Readmission Rates

Outcome	Change in Rate per Month, Slope (95% CI), %^a^	At the Start of the Period, Change in Slope (95% CI), %	*P* Value for Change in Slope
Acute myocardial infarction			
In-hospital mortality			
Pre-HRRP (January 2006 to March 2010)	−0.021 (−0.027 to −0.015)		
Post-HRRP announcement (April 2010 to September 2012)	−0.001 (−0.010 to 0.009)	0.020 (0.006 to 0.035)	.01^b^
HRRP penalties (October 2012 to December 2014)	−0.021 (−0.036 to −0.005)	−0.020 (−0.043 to 0.003)	.08
30-d postdischarge mortality			
Pre-HRRP (January 2006 to March 2010)	0.002 (−0.001 to 0.006)		
Post-HRRP announcement (April 2010 to September 2012)	−0.001 (−0.008 to 0.005)	−0.004 (−0.013 to 0.005)	.42
HRRP penalties (October 2012 to December 2014)	0.000 (−0.010 to 0.010)	0.001 (−0.013 to 0.016)	.86
30-d postdischarge readmission^c^			
Pre-HRRP (January 2006 to March 2010)	−0.001 (−0.007 to 0.005)		
Post-HRRP announcement (April 2010 to September 2012)	−0.059 (−0.069 to −0.05)	−0.058 (−0.072 to −0.044)	<.001
HRRP penalties (October 2012 to December 2014)	−0.042 (−0.057 to −0.027)	0.018 (−0.005 to 0.040)	.12
Heart failure			
In-hospital mortality			
Pre-HRRP (January 2006 to March 2010)	−0.014 (−0.018 to −0.010)		
Post-HRRP announcement (April 2010 to September 2012)	−0.002 (−0.008 to 0.005)	0.012 (0.003 to 0.022)	.01^b^
HRRP penalties (October 2012 to December 2014)	−0.014 (−0.025 to −0.004)	−0.013 (−0.028 to 0.003)	.10
30-d postdischarge mortality			
Pre-HRRP (January 2006 to March 2010)	0.004 (0.000 to 0.007)		
Post-HRRP announcement (April 2010 to September 2012)	0.010 (0.005 to 0.016)	0.006 (−0.002 to 0.015)	.11
HRRP penalties (October 2012 to December 2014)	0.006 (−0.003 to 0.015)	−0.005 (−0.018 to 0.009)	.49
30-d postdischarge readmission^c^			
Pre-HRRP (January 2006 to March 2010)	−0.001 (−0.006 to 0.005)		
Post-HRRP announcement (April 2010 to September 2012)	−0.085 (−0.094 to −0.075)	−0.084 (−0.098 to −0.070)	<.001
HRRP penalties (October 2012 to December 2014)	−0.022 (−0.037 to −0.006)	0.062 (0.040 to 0.085)	<.001
Pneumonia			
In-hospital mortality			
Pre-HRRP (January 2006 to March 2010)	−0.010 (−0.014 to −0.005)		
Post-HRRP announcement (April 2010 to September 2012)	−0.018 (−0.025 to −0.011)	−0.008 (−0.019 to 0.002)	.11
HRRP penalties (October 2012 to December 2014)	−0.023 (−0.035 to −0.011)	−0.005 (−0.022 to 0.012)	.55
30-d postdischarge mortality			
Pre-HRRP (January 2006 to March 2010)	0.005 (0.002 to 0.008)		
Post-HRRP announcement (April 2010 to September 2012)	−0.001 (−0.006 to 0.004)	−0.005 (−0.012 to 0.002)	.16
HRRP penalties (October 2012 to December 2014)	0.004 (−0.004 to 0.013)	0.005 (−0.007 to 0.017)	.42
30-d postdischarge readmission^c^			
Pre-HRRP (January 2006 to March 2010)	−0.005 (−0.010 to 0.000)		
Post-HRRP announcement (April 2010 to September 2012)	−0.052 (−0.060 to −0.044)	−0.047 (−0.059 to −0.035)	<.001
HRRP penalties (October 2012 to December 2014)	−0.014 (−0.027 to −0.001)	0.038 (0.019 to 0.057)	<.001

Abbreviation: HRRP, Hospital Readmissions Reduction Program.

^a^ Slopes represent the slope of the regression line in the corresponding period, representing the average change in mortality and readmission rates over a month in this period.

^b^ Significant, after applying Holm-Bonferroni adjustment to maintain a family-wide type I error rate of .05. (All *P* values for the outcomes of in-hospital and 30-day postdischarge mortality that are not marked “^b^” are not significant.)

^c^ *P* value threshold for significance for the secondary outcome of 30-day postdischarge readmission was .05.

In a nonparametric regression analysis, there were no periods of rising in-hospital or postdischarge mortality. Further, while readmission rates remained unchanged through 2010 (slope for monthly change, 0.000%; 95% CI, −0.006% to 0.005%; *P* for trend = .87), there was a negative deflection in monthly readmission rates in 2010 and readmission rates decreased by 0.051% per month after 2010 (95% CI −0.056% to −0.046%; *P* for trend <.001; *P* for change in slope <.001).

#### Heart Failure

**Figure 3.**
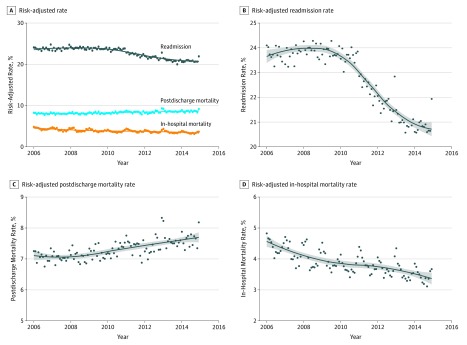
Time Trends in Risk-Adjusted Mortality and Readmissions for Heart Failure A, Trend plot for risk-adjusted monthly in-hospital mortality, postdischarge 30-day mortality, and 30-day readmission. Trend lines represent nonparametric locally weighted regression (Loess) lines. B, C, and D, Trend lines with expanded axes. The gray band represents the 95% confidence interval for trend lines.

Among patients hospitalized for HF, while both risk-adjusted in-hospital mortality and readmission rates decreased, risk-adjusted postdischarge mortality increased (Figure 3). The in-hospital mortality for HF decreased by 0.014% (95% CI, −0.018% to −0.010%) per month in the pre-HRRP period, with no significant changes in the post-HRRP announcement period, and a continued decrease in in-hospital mortality by 0.014% per month (−0.025% to −0.004%) in the HRRP penalty period. Risk-adjusted postdischarge mortality, in contrast, was increasing by 0.004% (95% CI, 0.000% to 0.007%) per month before the announcement of HRRP, and continued to increase both after HRRP announcement and the implementation of its penalties, without any changes in the slope of mortality rates at either time point (*P* > .05 for both) (Table). In contrast, there were no significant changes in risk-adjusted 30-day readmission rates in the pre-HRRP period, while the rates decreased significantly during both the post-HRRP announcement period (−0.085% per month; 95% CI, −0.094% to −0.075 per month) and HRRP penalty period (−0.022% per month; 95% CI, −0.037% to −0.006% per month) (Table).

In nonparametric regression analysis, we observed a change in trends of risk-adjusted postdischarge mortality before the announcement of the HRRP, which started as early as 2007. While there was no significant monthly change in mortality rates in 2006 (−0.013% per month; 95% CI, −0.031% to 0.005 per month; *P* for trend = .16), after 2007, mortality increased by 0.007% per month (95% CI, 0.006% to 0.009% per month; *P* for trend < .001; *P* for change in slope = .04). In contrast, readmission trends changed in 2010, the year of the announcement of the HRRP, without a significant change in monthly readmission rates before 2010 (−0.003% per month; 95% CI, −0.010% to 0.003% per month; *P* for trend = .33), but decreased by 0.060% per month after 2010 (95% CI, −0.066% to −0.055% per month; *P* for trend < .001; *P* for change in slope < .001).

#### Pneumonia

**Figure 4.**
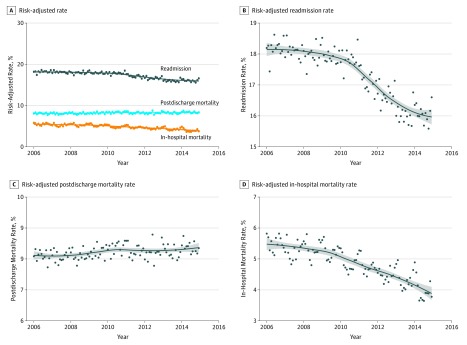
Time Trends in Risk-Adjusted Mortality and Readmissions for Pneumonia A, Trend plot for risk-adjusted monthly in-hospital mortality, postdischarge 30-day mortality, and 30-day readmission. Trend lines represent nonparametric locally weighted regression (Loess) lines. B, C, and D, Trend lines with expanded axes. The gray band represents the 95% confidence interval for trend lines.

Among those hospitalized with pneumonia, rates of risk-adjusted in-hospital mortality and readmission decreased, while postdischarge mortality increased (Figure 4). Monthly risk-adjusted in-hospital mortality rates decreased by 0.010% per month (95% CI, −0.014% to −0.005% per month) in the pre-HRRP period, and continued to decrease significantly after both the announcement of HRRP and the implementation of its penalties. In contrast, 30-day postdischarge mortality increased by 0.005% per month (95% CI, 0.002% to 0.008% per month) in the pre-HRRP period, with no significant changes in the post-HRRP announcement and HRRP penalty periods. In a pattern similar to that observed for AMI and HF, risk-adjusted readmission rates did not change significantly in the pre-HRRP period, but decreased by 0.052% per month (95% CI, −0.060% to −0.044% per month) in the post-HRRP announcement period and decreased by 0.014% per month (95% CI, −0.027% to −0.001% per month) in the HRRP penalty period (Table).

In a nonparametric regression analysis, over the study period, in-hospital mortality decreased by 0.014% per month (95% CI, −0.016% to −0.013% per month; *P* for trend < .001), but postdischarge mortality increased by 0.003% per month (95% CI, 0.001% to 0.004% per month; *P* for trend < .001), without any points of inflection. However, similar to the pattern observed for both AMI and HF, readmission trends changed in 2010, the year of the announcement of the HRRP. Before 2010, monthly readmission rates did not change significantly (slope for monthly change, −0.003%; 95% CI, −0.010% to 0.003%; *P* for trend = .33), but decreased by 0.060% per month after 2010 (95% CI, −0.066% to −0.055% per month; *P* for trend < .001; *P* for change in slope < .001).

**Figure 5.**
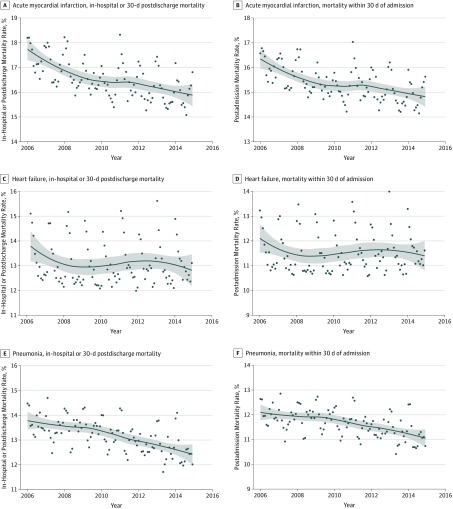
Time Trends in Risk-Adjusted Hospitalization-Related Mortality A, C, and E, Trend plots for risk-adjusted monthly rates of mortality either during hospitalization or within 30 days of discharge following hospitalization for acute myocardial infarction (A), heart failure (C) and pneumonia (E). B, D, and F, Trend plots for corresponding trends for mortality within 30 days of admission. Trend lines represent nonparametric locally weighted regression (Loess) lines. The gray band represents the 95% confidence interval for trend lines.

Our findings were consistent across several sensitivity analyses. Specifically, the patterns in unadjusted and risk-adjusted monthly rates were similar for our primary mortality and readmission outcomes (eFigures 7 through 9 and eTable 4 in the Supplement). Moreover, although there was a clear seasonal variation in rates of both mortality and readmission for all conditions, temporal trends were consistent in analyses that explicitly accounted for this variation (eFigure 10 in the Supplement). Finally, in an evaluation of the overall mortality related to hospitalizations, accounting for deaths both during and after a hospitalization event, risk-adjusted mortality decreased for all 3 conditions, which was most prominent in AMI (Figure 5). Moreover, for both outcomes, none of the observed trends were associated with changes at either of 2 time points associated with the HRRP (eTable 5 in the Supplement).

## Discussion

Following the announcement of the HRRP and the implementation of its penalties—a period with large decreases in readmission rates—in-hospital mortality among Medicare beneficiaries hospitalized for all 3 targeted conditions decreased without any associated inflections in postdischarge 30-day mortality. While postdischarge mortality for HF and pneumonia rose over the study period, these increases began in 2007 and 2006, respectively, over 3 years before the announcement of the HRRP and 5 years before the implementation of its associated financial penalties. Moreover, neither the announcement of the HRRP nor its implementation was associated with an increase in the changes in postdischarge mortality rates. These findings from the overall Medicare fee-for-service population do not support an assertion that either the HRRP or its associated declines in readmission rates are associated with increasing mortality, because mortality rates either did not change or started to increase well before the HRRP passage and any decrease in readmissions.

Our study extends the prior literature in several ways. It addresses the concerns of an increase in mortality after the implementation of the HRRP.^5,7,8,26^ Our use of the entire fee-for-service Medicare sample quantifies temporal mortality trends in the population targeted by the HRRP and thereby specifically overcomes limitations of nonrepresentative sampling, random variation, and small sample size in other studies.^7,27^ Specifically, a study by Gupta et al^7^ found an increase in mortality in postdischarge mortality among Medicare beneficiaries included from hospitals participating in the American Heart Association’s Get With The Guidelines–Heart Failure registry that was temporally associated with the implementation of the HRRP. However, their study population represents a selected group of Medicare beneficiaries compiled from voluntary reporting by hospitals.^28,29^ Therefore, the completeness of the data from participating hospitals as well as their representativeness to all Medicare beneficiaries nationally is unclear. Our study replicates some of the analyses in this study using the entire fee-for-service Medicare data and argues against the generalizability of their observations. Our study findings are also supported by a recent report from the Medicare Payment Advisory Commission, a nonpartisan federal agency, which also found no association between the HRRP and patient mortality with any of 3 conditions targeted in the program.^18^

Moreover, our study was not limited to hospitals that reduced readmissions, but addressed the potential for harm across all hospitals, thereby obviating the concerns about increases in mortality at hospitals that did not achieve reductions in readmissions but still caused harm to patients.^8,26^ Further, our assessment of both in-hospital and postdischarge mortality across all 3 of the HRRP conditions allows for an assessment of systematic changes in practices that are associated with signals for harm to patients. Findings from our study are particularly reassuring as the CMS considers expanding the HRRP to include a hospital-wide readmission measure with the goal of reducing readmissions for the entire spectrum of patients.^28^

A notable finding is that postdischarge mortality among patients with pneumonia and HF has been rising since 2007. Prior studies and publicly available data suggest a similar trend.^30,31,32^ For HF, this represents a change from improvements observed in prior decades.^33^ There are no clear explanations for this change. For both HF and pneumonia, we found an increase in the coded severity of illness as well as a decrease in the number of hospitalizations. Therefore, the increasing complexity of patients may manifest with an increase in early postdischarge mortality, particularly because risk adjustment may not adequately account for all changes in illness severity over time. Further, the decreasing in-hospital mortality despite such an increase in coded severity argues that there may be alternative factors driving this trend in postdischarge mortality. Notably, there has been a concomitant decrease in in-hospital mortality for both conditions, and accounting for this decrease in the assessment of hospitalization-related mortality attenuates the increased mortality observed in the postdischarge setting. This also suggest that while fewer patients died during hospitalization over time, the actual improvement in patient outcomes may be less substantial.

We also found that the temporal trends for unadjusted readmission as well as mortality rates were similar to those for their corresponding risk-adjusted rates. These findings suggest that improvements in readmission rates as well as increases in mortality rates do not merely represent a manifestation in coded severity of illness,^34^ but rather true trends in these outcomes.

### Limitations

Our analyses should be interpreted in light of several limitations. Our study does not specifically evaluate other factors that may be underlying secular trends in mortality, and only addresses these trends in relation to the HRRP. We also do not evaluate patterns in mortality occurring in those where patients receive care only in the outpatient setting, as these patients do not directly fall under the purview of the HRRP. The findings from our study are therefore not generalizable to such patients. Our use of an interrupted time series framework is effective in identifying discontinuities resulting from changes in health policy, but does not imply any causal effect of the HRRP on either readmissions or mortality.^35^ Further, Medicare claims data do not routinely include information on either the cause of death or end-of-life care, limiting our ability to further assess the cause of the observed patterns of mortality. We used comorbidities identified in administrative claims for risk adjustment, which differ from risk factors used in some other studies.^7^ However, this is unlikely to affect our findings because our risk-adjustment approach is the same as that used by CMS and has been validated previously for both mortality and readmission outcomes.^6^ Moreover, the overall trends in both the unadjusted and risk-adjusted analyses are consistent.

## Conclusions

Among fee-for-service elderly Medicare beneficiaries, there was no evidence for an increase in in-hospital or postdischarge mortality associated with either the HRRP announcement or implementation—a period with substantial reductions in readmissions. The improvement in readmission was therefore not associated with any increase in in-hospital or 30-day postdischarge mortality.

## Notes

Supplement. **eMethods.** Patient Selection **eFigure 1.** Patient Flowsheet: 30-day Postdischarge Readmission and Mortality Outcomes in Acute Myocardial Infarction **eFigure 2. **Patient Flowsheet: 30-day Postdischarge Readmission and Mortality Outcomes in Heart Failure **eFigure 3. **Patient Flowsheet: 30-day Postdischarge Readmission and Mortality Outcomes in Pneumonia **eFigure 4.** Patient Flowsheet: In-hospital Mortality in Acute Myocardial Infarction (AMI) **eFigure 5.** Patient Flowsheet: In-hospital Mortality in Heart Failure **eFigure 6.** Patient Flowsheet: In-hospital Mortality in Pneumonia **eFigure 7.** Time Trends in Unadjusted Rates of Mortality and Readmission, Acute Myocardial Infarction **eFigure 8.** Time Trends in Unadjusted Rates of Mortality and Readmission for Heart Failure **eFigure 9.** Time Trends in Unadjusted Rates of Mortality and Readmission for Pneumonia **eFigure 10.** Time Trends in Risk-adjusted Mortality and Readmission after Accounting for Seasonal Variation **eTable 1.** Characteristics of Patients Hospitalized for Acute Myocardial Infarction, By Calendar-Year **eTable 2.** Characteristics of Patients Hospitalized for Heart Failure, By Calendar-Year **eTable 3.** Characteristics of Patients Hospitalized for Pneumonia, By Calendar-Year **eTable 4.** Interrupted Time Series for Unadjusted In-hospital Mortality, and 30-day Unadjusted Postdischarge Mortality and Readmission Rates **eTable 5. **Interrupted Time Series for Risk-Adjusted In-hospital/30-day Postdischarge Mortality and 30-day Post-admission Mortality
