# Supplementary material for: Association of the Hospital Readmissions Reduction Program With Mortality During and After Hospitalization for Acute Myocardial Infarction, Heart Failure, and Pneumonia
Source: JAMA Netw Open. 2018 Sep 28;1(5):e182777. doi: 10.1001/jamanetworkopen.2018.2777 (PMC6324473; doi:10.1001/jamanetworkopen.2018.2777)
Supplement: Supplement. — eMethods. Patient Selection eFigure 1. Patient Flowsheet: 30-day Postdischarge Readmission and Mortality Outcomes in Acute Myocardial Infarction eFigure 2. Patient Flowsheet: 30-day Postdischarge Readmission and Mortality Outcomes in Heart Failure eFigure 3. Patient Flowsheet: 30-day Postdischarge Readmission and Mortality Outcomes in Pneumonia eFigure 4. Patient Flowsheet: In-hospital Mortality in Acute Myocardial Infarction (AMI) eFigure 5. Patient Flowsheet: In-hospital Mortality in Heart Failure eFigure 6. Patient Flowsheet: In-hospital Mortality in Pneumonia eFigure 7. Time Trends in Unadjusted Rates of Mortality and Readmission, Acute Myocardial Infarction eFigure 8. Time Trends in Unadjusted Rates of Mortality and Readmission for Heart Failure eFigure 9. Time Trends in Unadjusted Rates of Mortality and Readmission for Pneumonia eFigure 10. Time Trends in Risk-adjusted Mortality and Readmission after Accounting for Seasonal Variation eTable 1. Characteristics of Patients Hospitalized for Acute Myocardial Infarction, By Calendar-Year eTable 2. Characteristics of Patients Hospitalized for Heart Failure, By Calendar-Year eTable 3. Characteristics of Patients Hospitalized for Pneumonia, By Calendar-Year eTable 4. Interrupted Time Series for Unadjusted In-hospital Mortality, and 30-day Unadjusted Postdischarge Mortality and Readmission Rates eTable 5. Interrupted Time Series for Risk-Adjusted In-hospital/30-day Postdischarge Mortality and 30-day Post-admission Mortality [file jamanetwopen-1-e182777-s001.pdf]

Supplementary Online Content

Khera R, Dharmarajan K, Wang Y, et al. Association of the hospital readmissions reduction program with mortality during and after hospitalization for acute myocardial infarction, heart failure, and pneumonia. *JAMA Netw Open*. 2018;1(5):e182777. doi:10.1001/jamanetworkopen.2018.2777

eMethods. Patient Selection

eFigure 1. Patient Flowsheet: 30-day Postdischarge Readmission and Mortality Outcomes in Acute Myocardial Infarction

eFigure 2. Patient Flowsheet: 30-day Postdischarge Readmission and Mortality Outcomes in Heart Failure

eFigure 3. Patient Flowsheet: 30-day Postdischarge Readmission and Mortality Outcomes in Pneumonia

eFigure 4. Patient Flowsheet: In-hospital Mortality in Acute Myocardial Infarction (AMI)

eFigure 5. Patient Flowsheet: In-hospital Mortality in Heart Failure

eFigure 6. Patient Flowsheet: In-hospital Mortality in Pneumonia

eFigure 7. Time Trends in Unadjusted Rates of Mortality and Readmission, Acute Myocardial Infarction

eFigure 8. Time Trends in Unadjusted Rates of Mortality and Readmission for Heart Failure

eFigure 9. Time Trends in Unadjusted Rates of Mortality and Readmission for Pneumonia

eFigure 10. Time Trends in Risk-adjusted Mortality and Readmission after Accounting for Seasonal Variation

eTable 1. Characteristics of Patients Hospitalized for Acute Myocardial Infarction, By Calendar-Year

eTable 2. Characteristics of Patients Hospitalized for Heart Failure, By Calendar-Year

eTable 3. Characteristics of Patients Hospitalized for Pneumonia, By Calendar-Year

eTable 4. Interrupted Time Series for Unadjusted In-hospital Mortality, and 30-day Unadjusted Postdischarge Mortality and Readmission Rates

eTable 5. Interrupted Time Series for Risk-Adjusted In-hospital/30-day Postdischarge Mortality and 30-day Post-admission Mortality

© 2018 Khera R et al. *JAMA Network Open*.

This supplementary material has been provided by the authors to give readers additional information about their work.

© 2018 Khera R et al. *JAMA Network Open*.

eMethods. Patient Selection

In the current study, patient cohorts used in assessing readmission and mortality measures of the Centers for Medicare and Medicaid Services (CMS) were used to define the study population for the different outcomes. This ensures that our observations are consistent with those reported from the CMS data. Specifically, the 30-day readmission outcome was used to define the study population for both the 30-day readmission outcomes and the 30-day post-discharge mortality. The patient selection flowsheet for these outcomes are presented in eFigures 1-3. Further, the 30-day post-admission mortality measure was used to define both in-hospital and 30-day post-admission outcomes. The selection criteria for in-hospital mortality for each of the three outcomes are presented in eFigures 4-6. The 30-day post-admission mortality, a secondary outcome in our study, additionally excluded patients that did not have information on 30-day follow up and randomly selected one hospitalization if there were multiple hospitalizations for an individual in a given year. These selection criteria are specific to how the 30-day post-admission mortality outcome is defined in the CMS measures. Finally, the cohort that defined in-hospital mortality was followed for 30 days post-discharge among all those who survived hospitalization to evaluate the combined outcome of in-hospital or 30-day post-discharge mortality.

© 2018 Khera R et al. *JAMA Network Open*.

eFigure 1. Patient Flowsheet: 30-day Postdischarge Readmission and Mortality Outcomes in Acute Myocardial Infarction

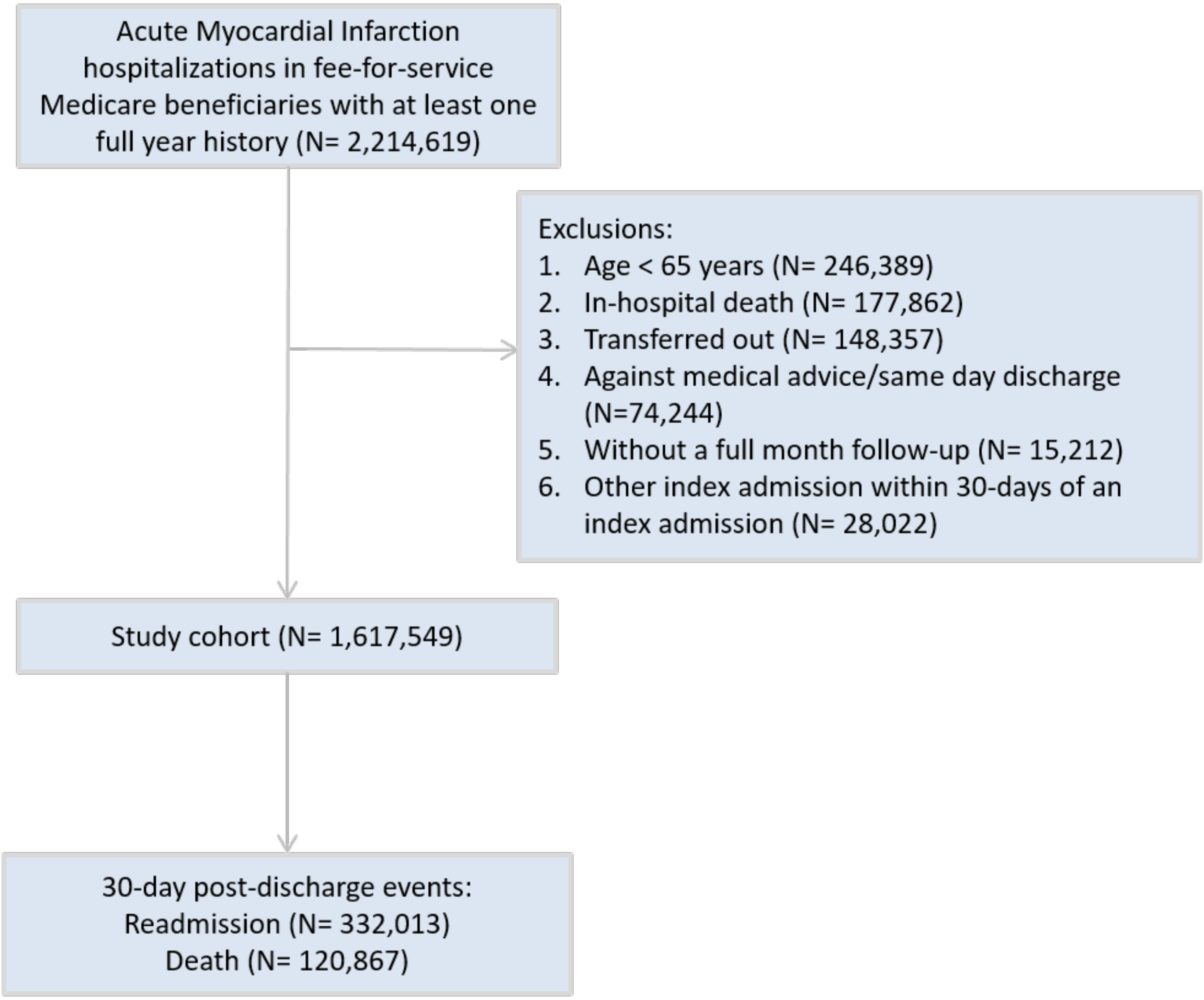

© 2018 Khera R et al. *JAMA Network Open*.

eFigure 2. Patient Flowsheet: 30-day Postdischarge Readmission and Mortality Outcomes in Heart Failure

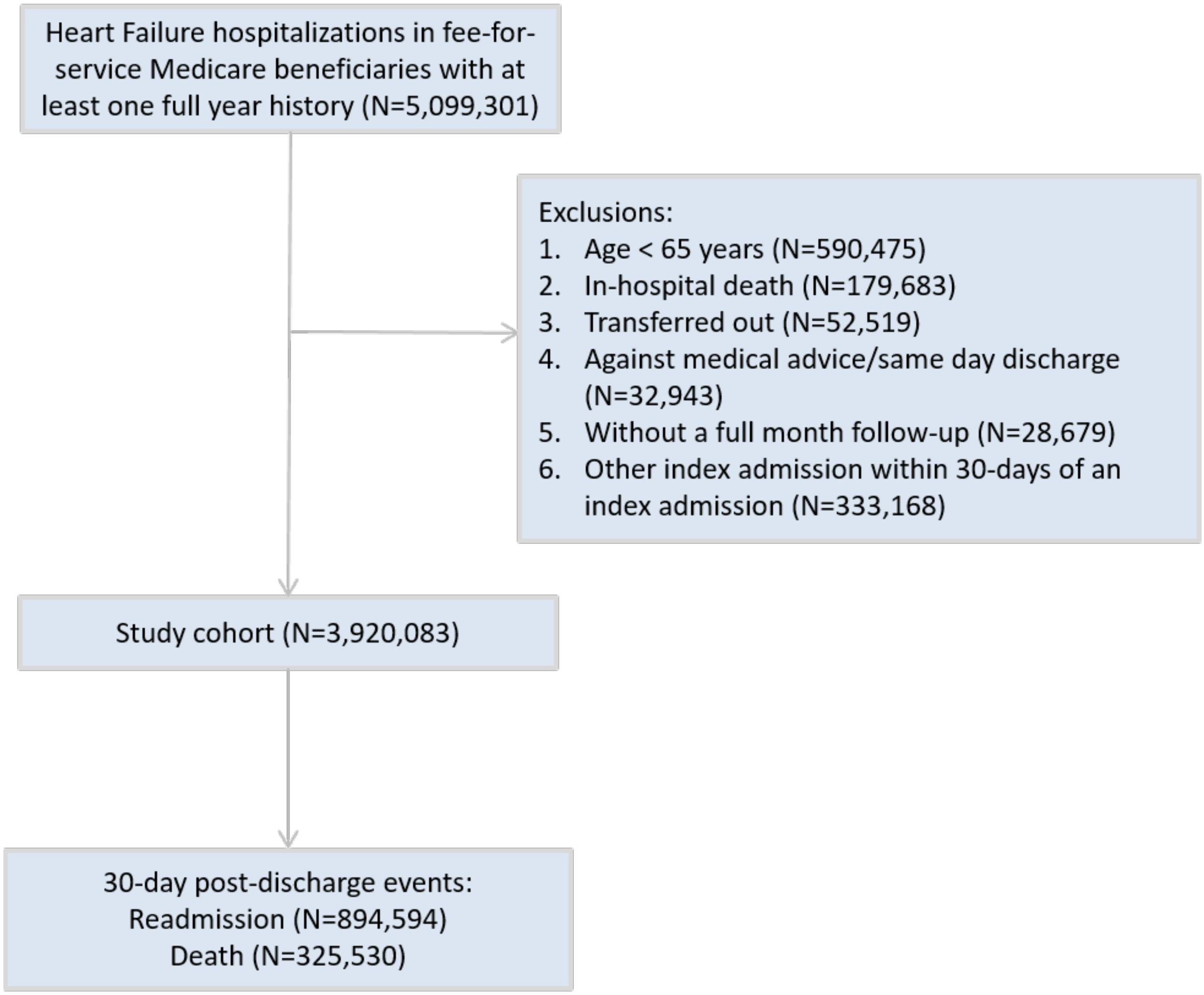

© 2018 Khera R et al. *JAMA Network Open*.

eFigure 3. Patient Flowsheet: 30-day Postdischarge Readmission and Mortality Outcomes in Pneumonia

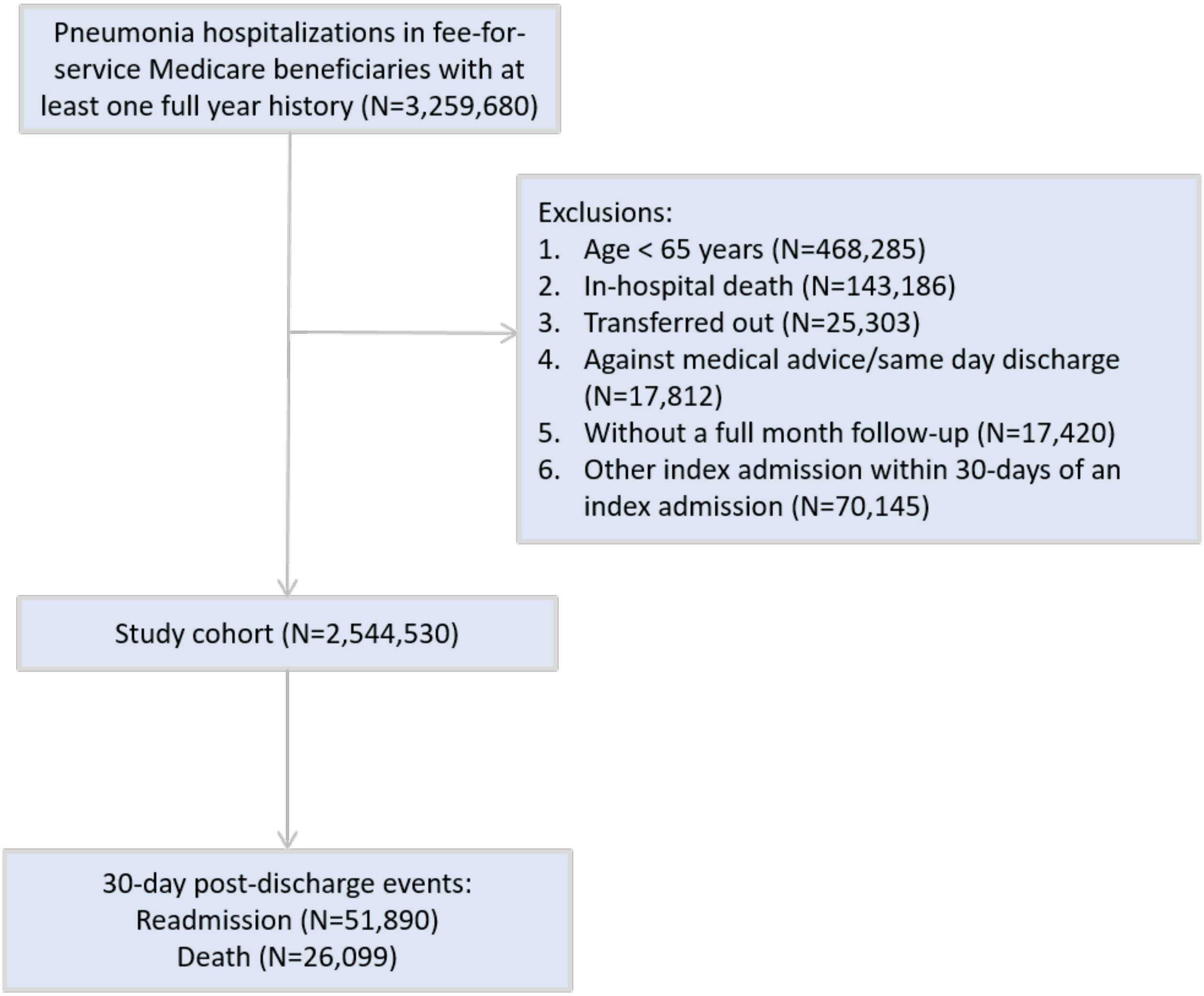

© 2018 Khera R et al. *JAMA Network Open*.

eFigure 4. Patient Flowsheet: In-hospital Mortality in Acute Myocardial Infarction (AMI)

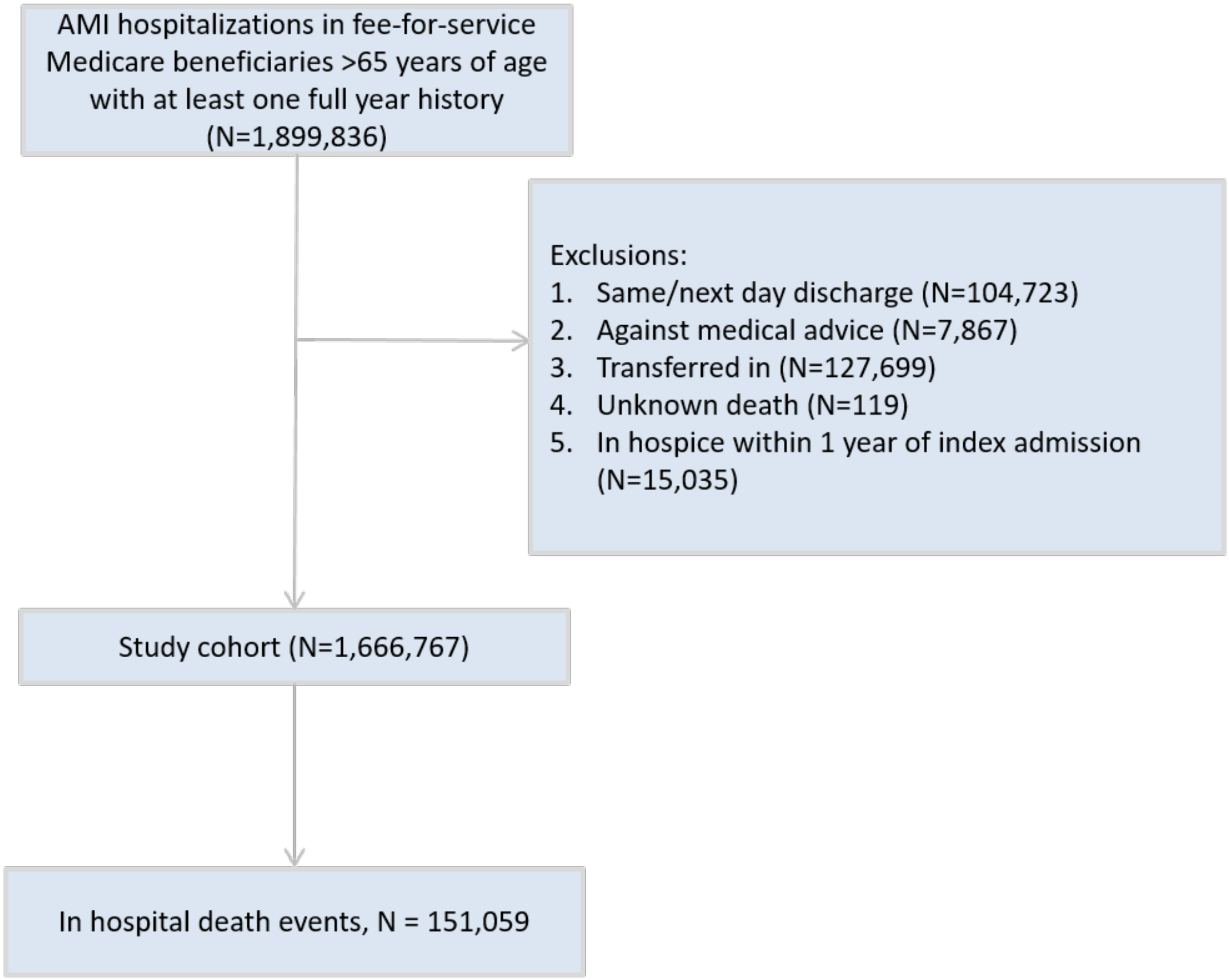

© 2018 Khera R et al. *JAMA Network Open*.

eFigure 5. Patient Flowsheet: In-hospital Mortality in Heart Failure

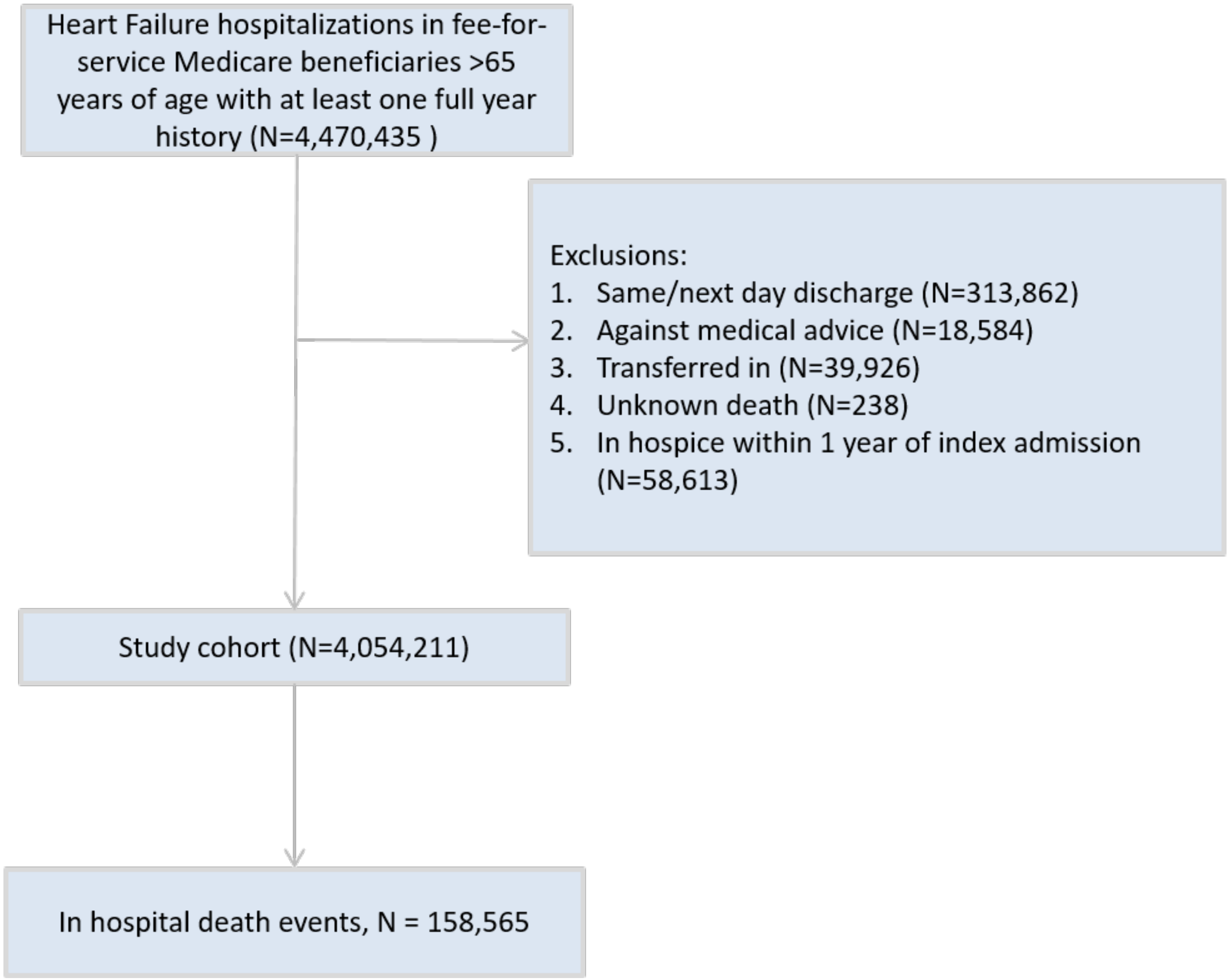

© 2018 Khera R et al. *JAMA Network Open*.

eFigure 6. Patient Flowsheet: In-hospital Mortality in Pneumonia

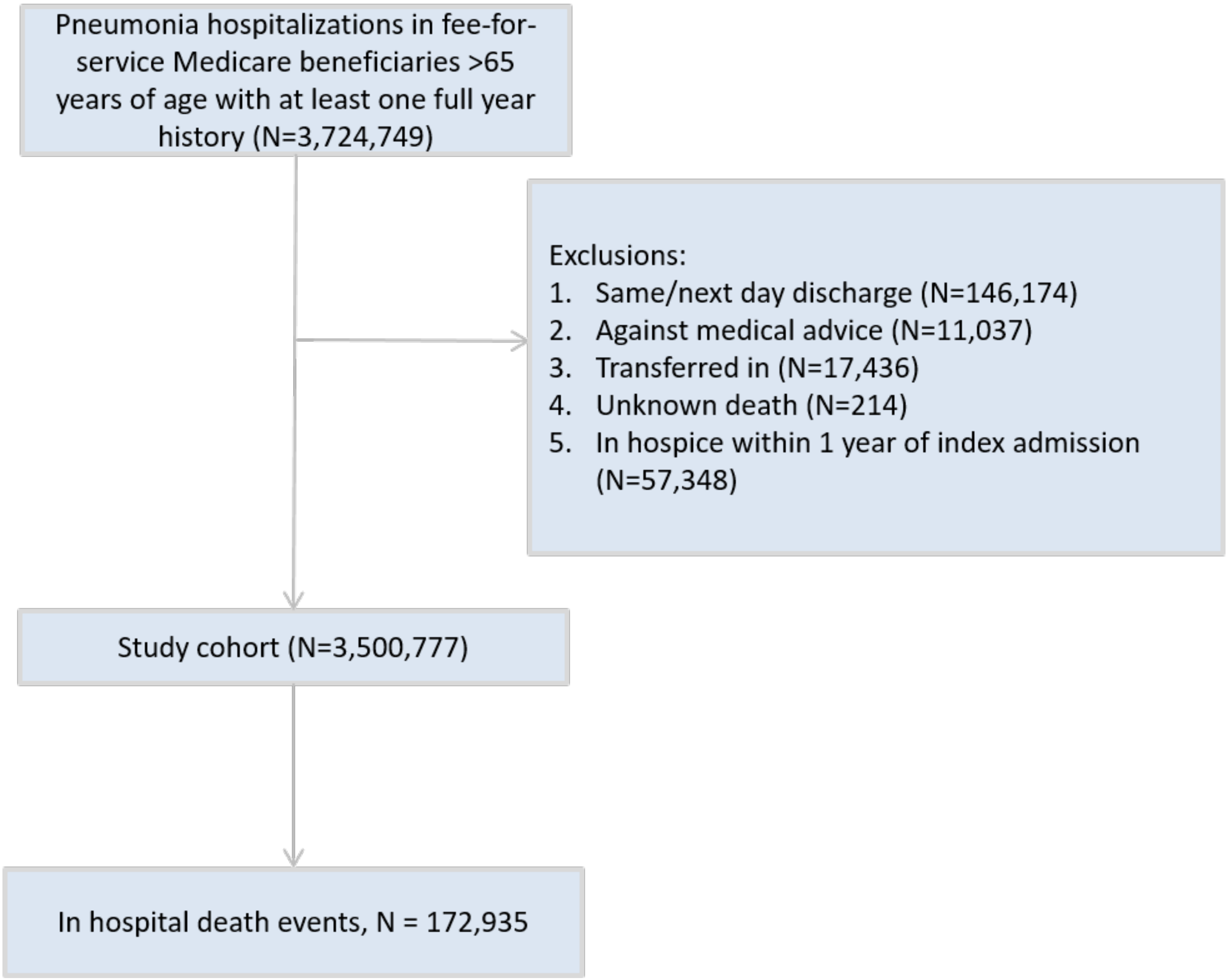

© 2018 Khera R et al. *JAMA Network Open*.

eFigure 7. Time Trends in Unadjusted Rates of Mortality and Readmission, Acute Myocardial Infarction

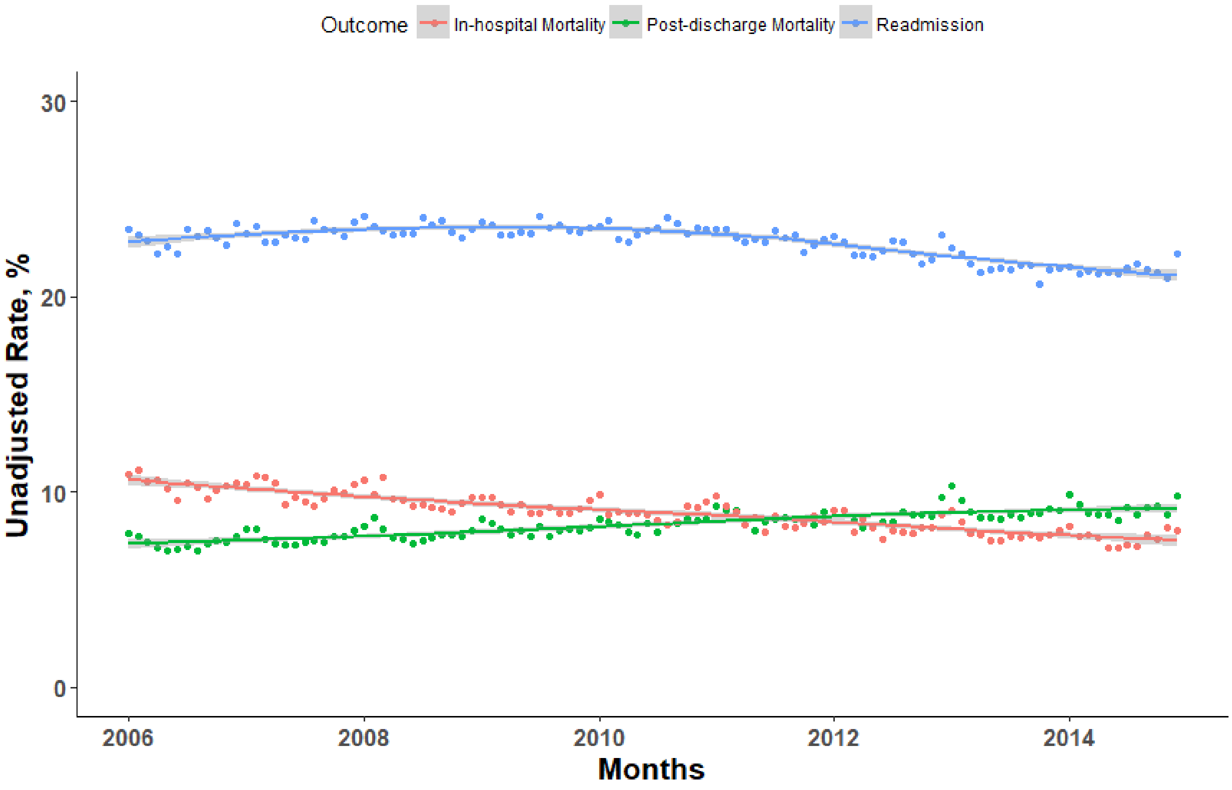

Lines represent Loess curves and their 95% confidence intervals.

© 2018 Khera R et al. *JAMA Network Open*.

eFigure 8. Time Trends in Unadjusted Rates of Mortality and Readmission for Heart Failure

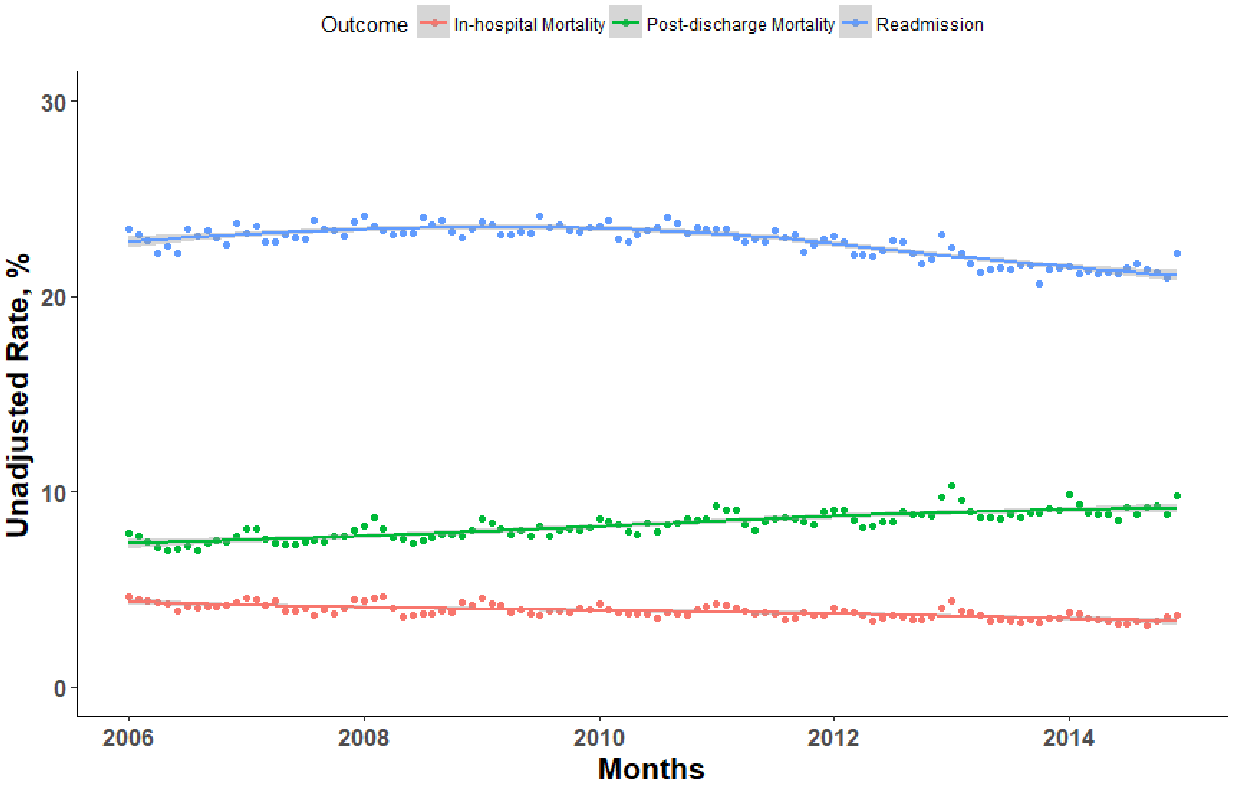

Lines represent Loess curves and their 95% confidence intervals.

© 2018 Khera R et al. *JAMA Network Open*.

eFigure 9. Time Trends in Unadjusted Rates of Mortality and Readmission for Pneumonia

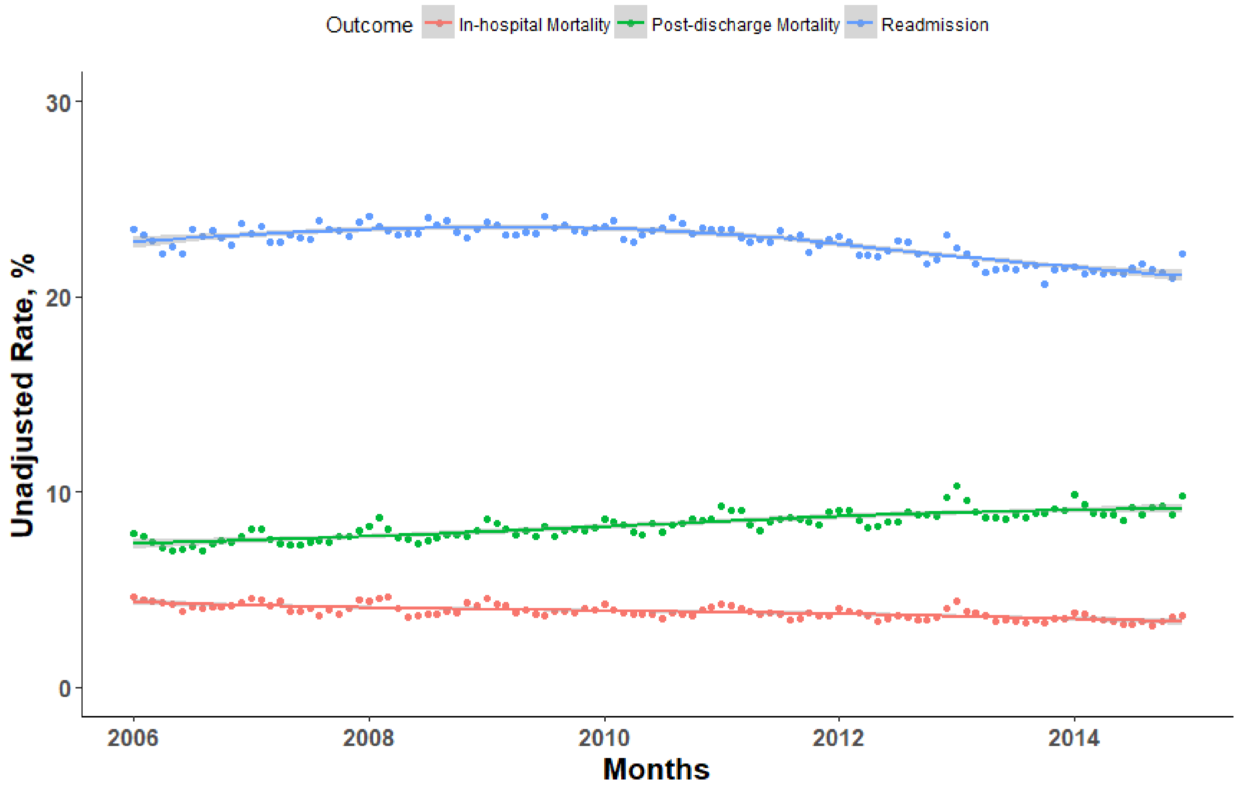

Lines represent Loess curves and their 95% confidence intervals.

© 2018 Khera R et al. *JAMA Network Open*.

eFigure 10. Time Trends in Risk-adjusted Mortality and Readmission after Accounting for Seasonal Variation

10A. AMI Risk-adjusted In-hospital Mortality

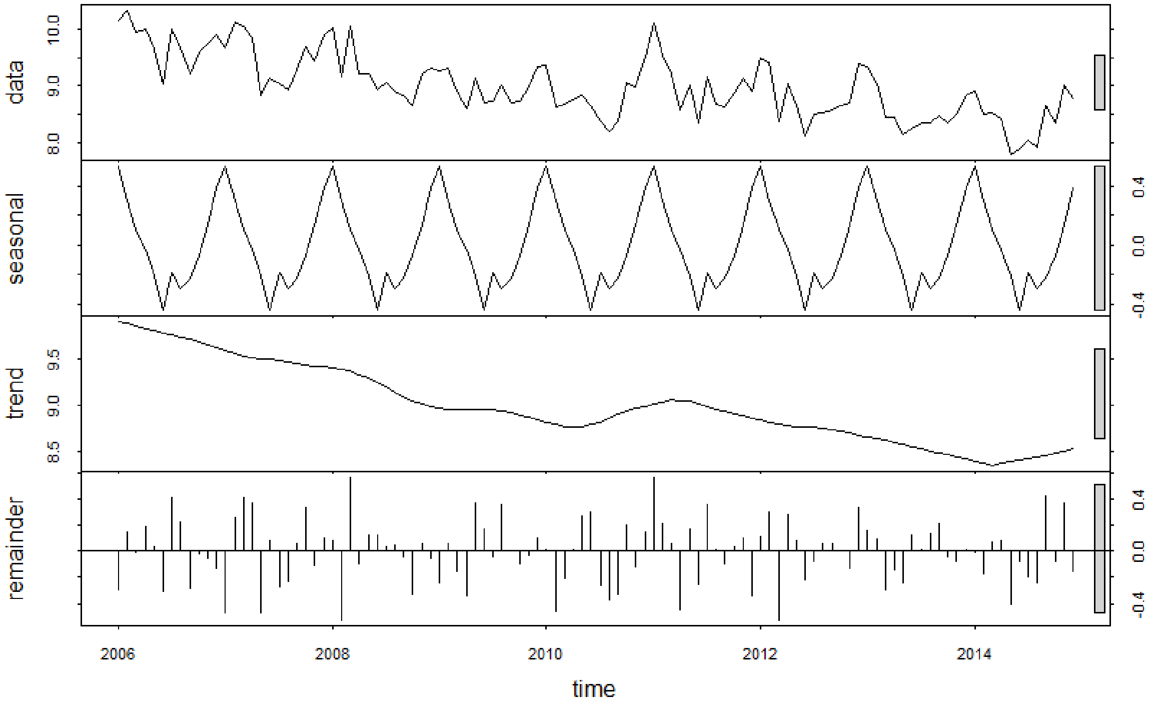

© 2018 Khera R et al. *JAMA Network Open*.

10B. AMI 30-day Risk-adjusted Postdischarge Mortality

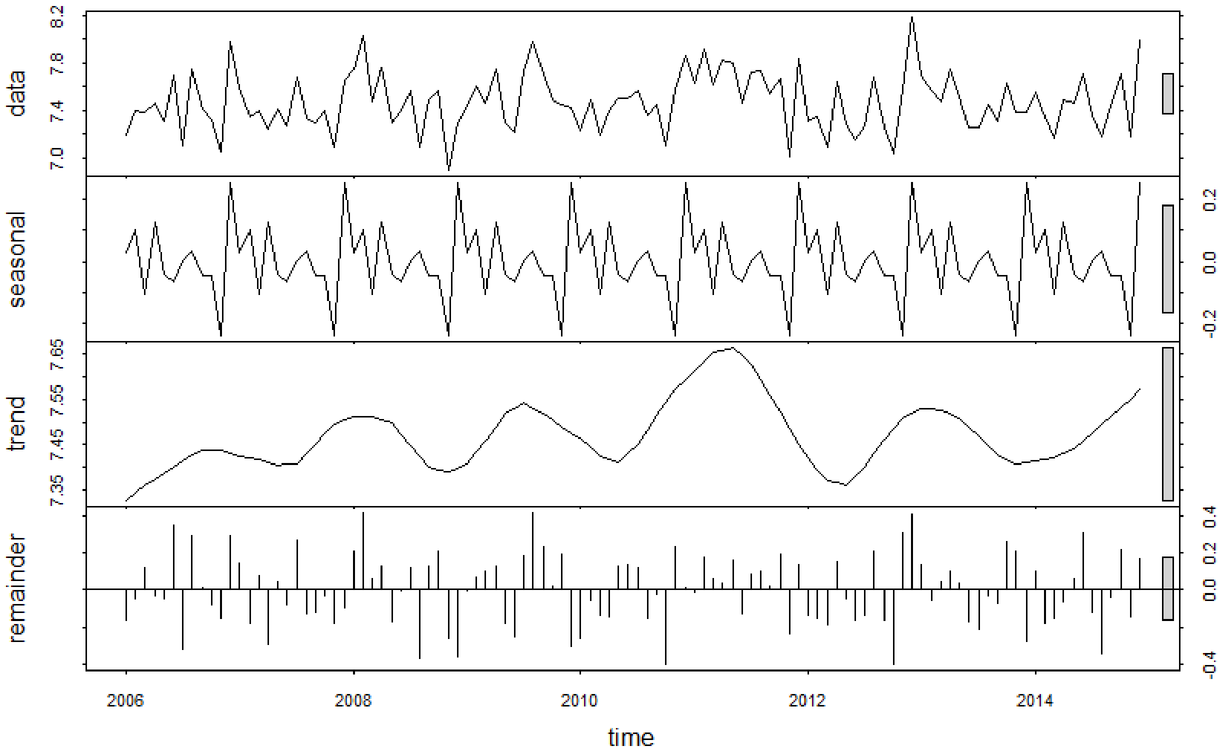

© 2018 Khera R et al. *JAMA Network Open*.

10C. AMI 30-day Risk-adjusted Readmission

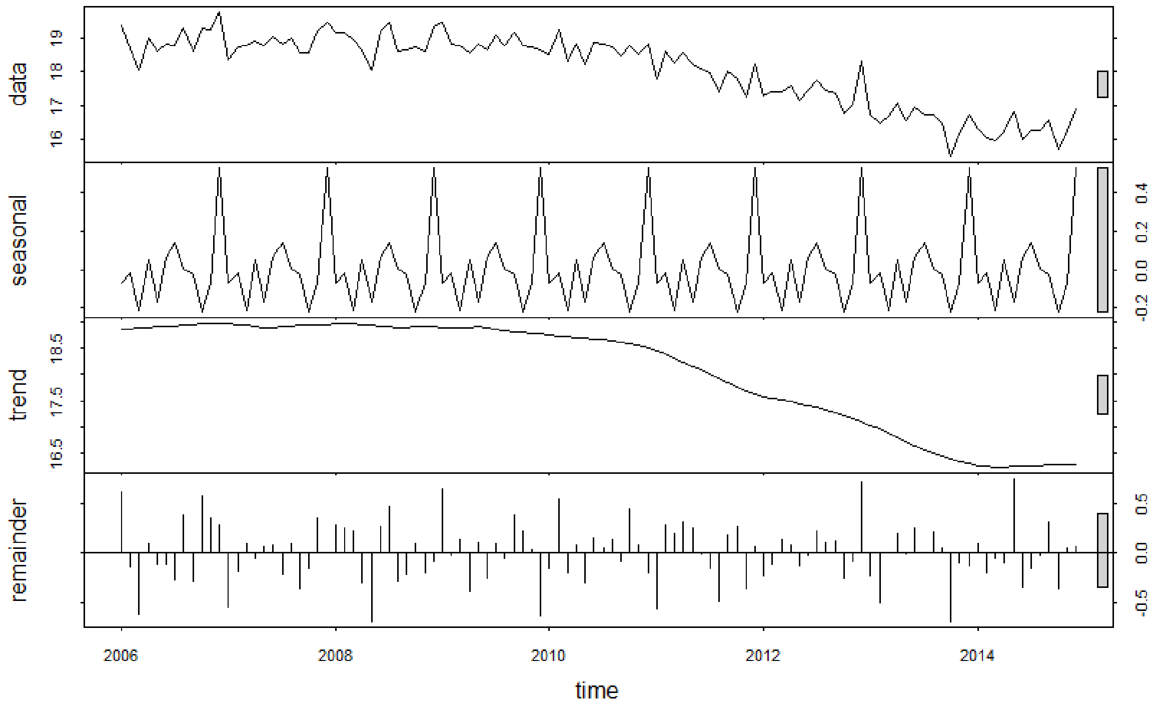

© 2018 Khera R et al. *JAMA Network Open*.

10D. Heart Failure In-hospital Mortality

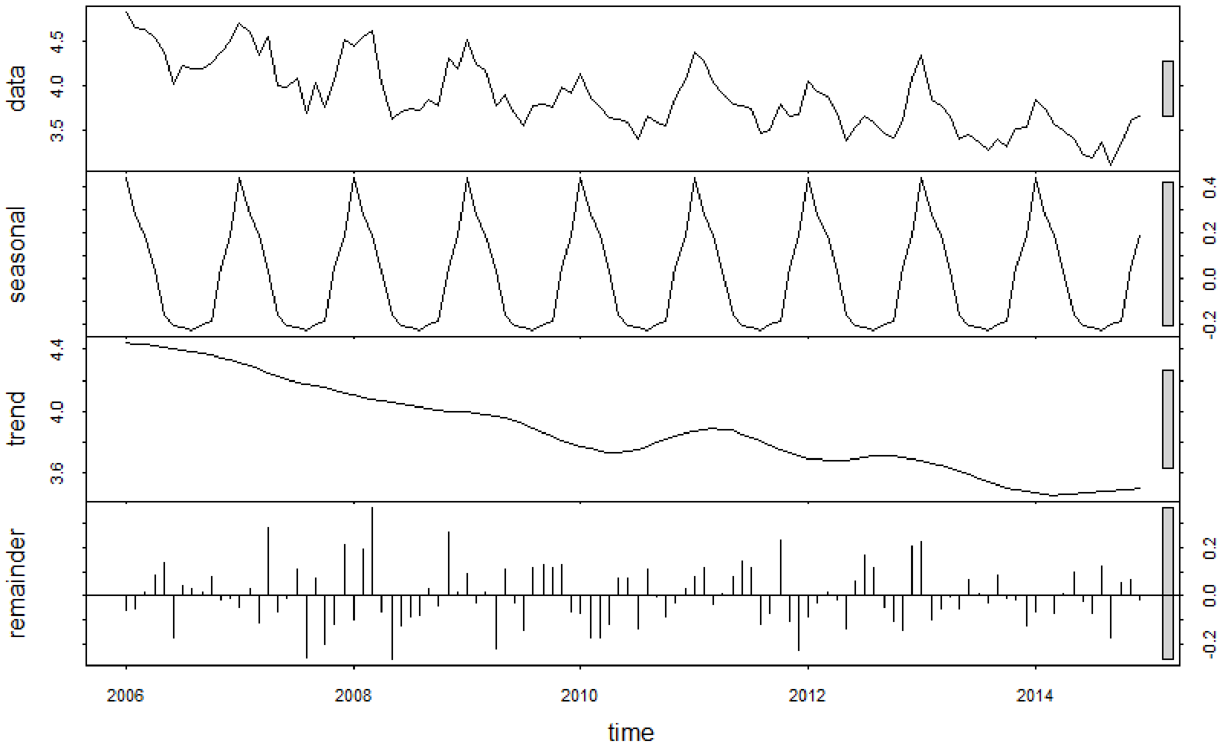

© 2018 Khera R et al. *JAMA Network Open*.

10E. Heart Failure 30-day Risk-adjusted Postdischarge Mortality

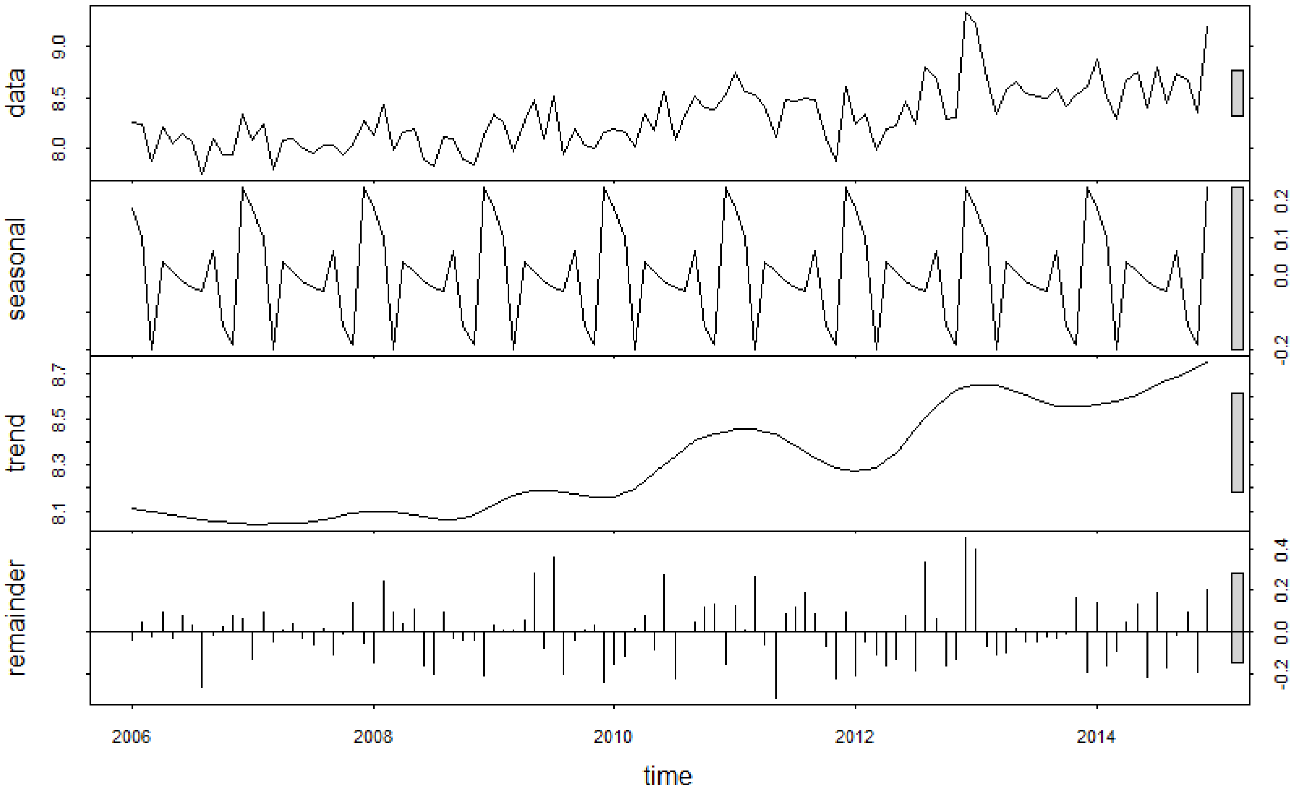

© 2018 Khera R et al. *JAMA Network Open*.

10F. Heart Failure 30-day Risk-adjusted Readmission

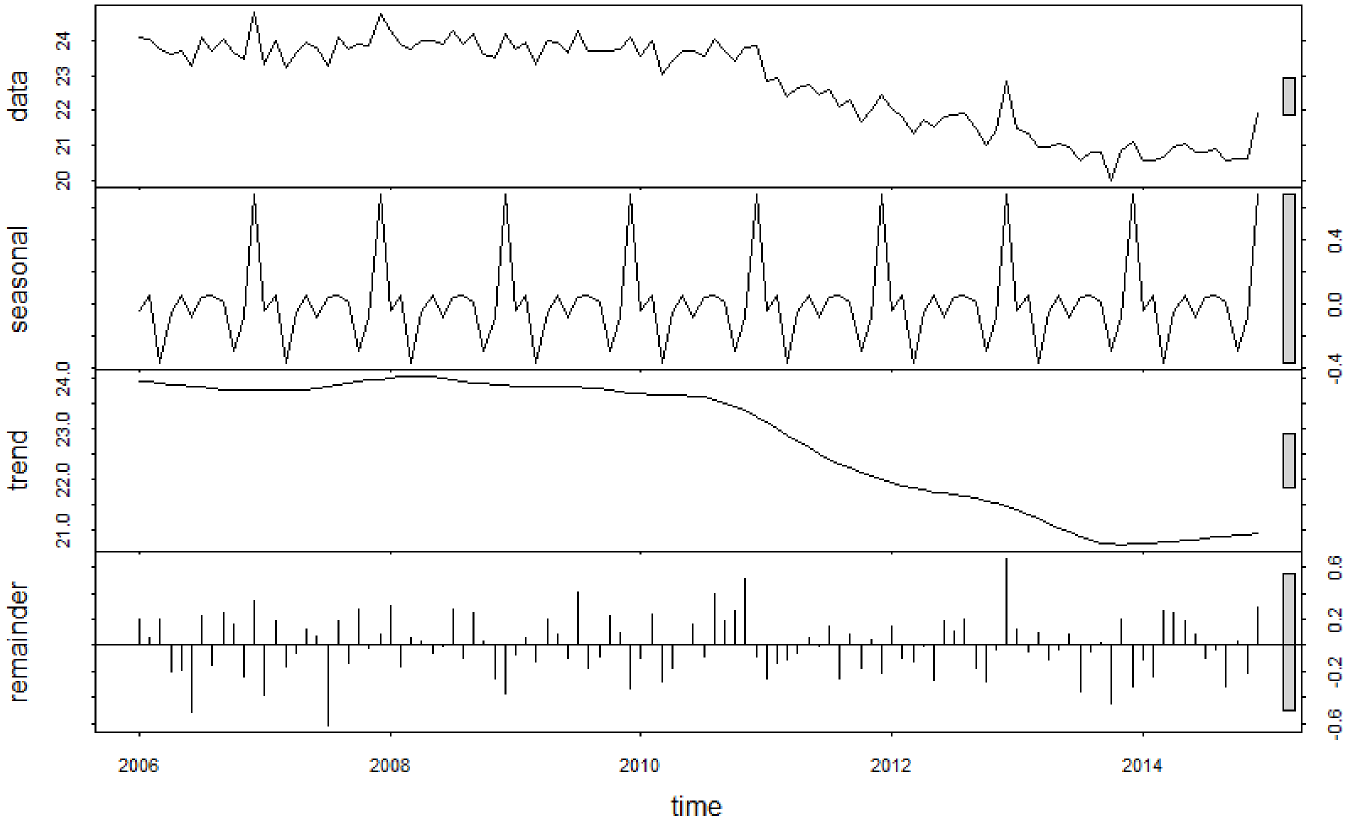

© 2018 Khera R et al. *JAMA Network Open*.

10G. Pneumonia Risk-adjusted In-hospital Mortality

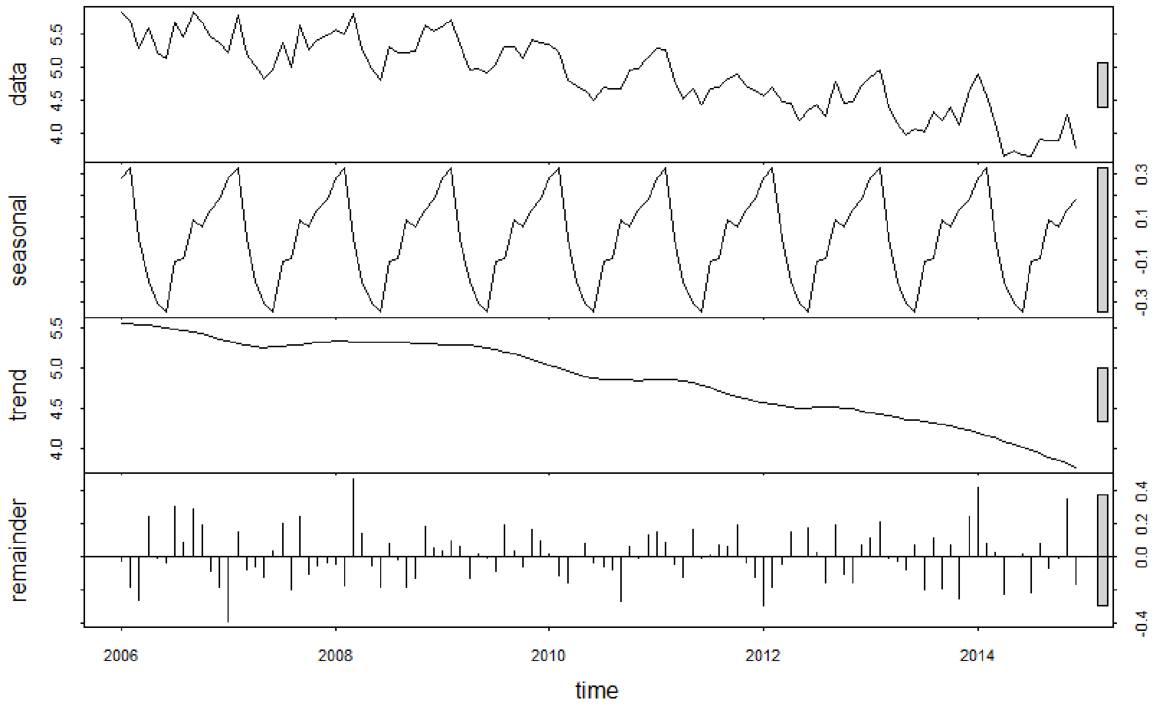

© 2018 Khera R et al. *JAMA Network Open*.

10H. Pneumonia 30-day Risk-adjusted Postdischarge Mortality

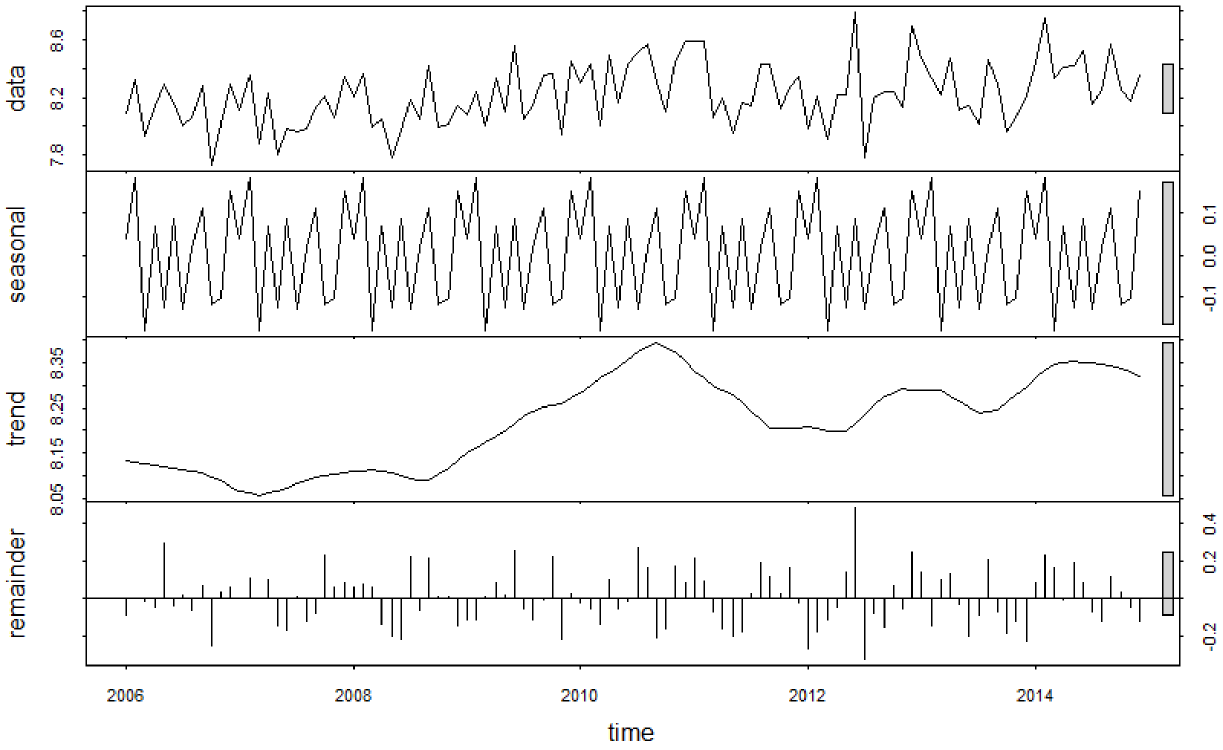

© 2018 Khera R et al. *JAMA Network Open*.

10I. Pneumonia 30-day Risk-adjusted Readmission

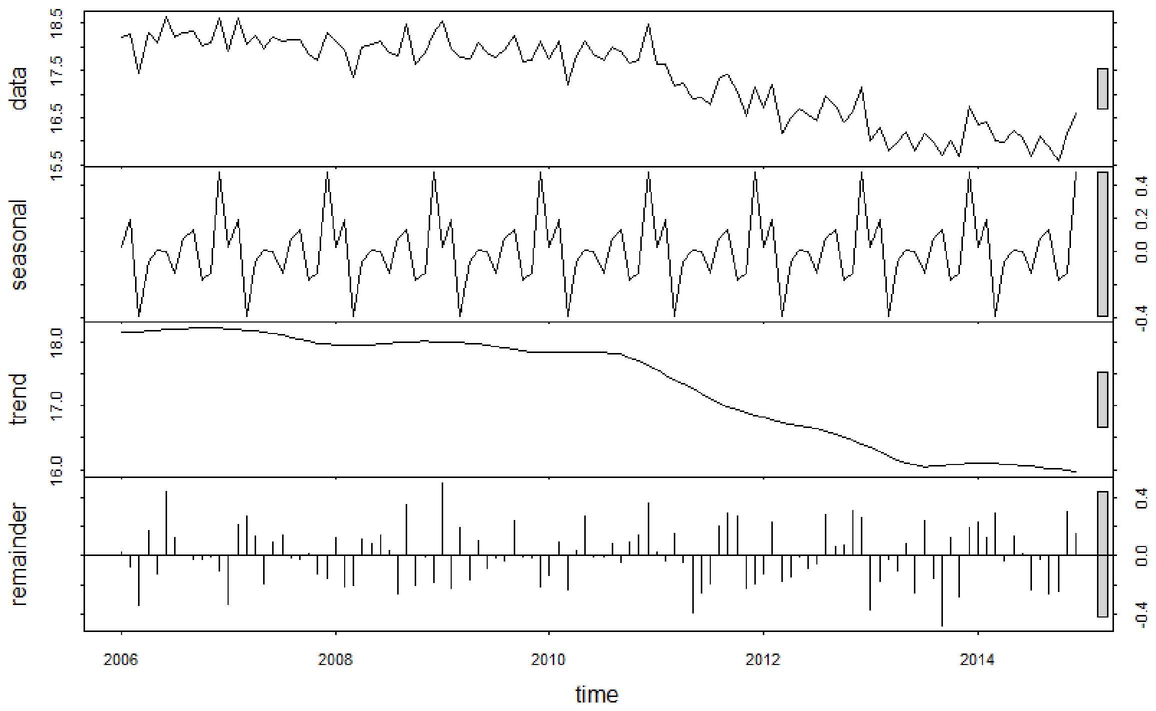

An iterative filtering procedure decomposed the time series into three components – seasonal, trend, and random variation. The variation that occurs periodically represents the 'seasonal' component, the underlying time trend is then assigned using the Loess procedure and represents the 'trend' component, and finally, the random variation at each time point is the difference between the actual data value and the sum of 'seasonal' and 'trend' components at each time point ('remainder'). X-axes represent rates of mortality or readmissions. Gray bars on the right present a scale for these axes. AMI, acute myocardial infarction.

© 2018 Khera R et al. *JAMA Network Open*.

eTable 1. Characteristics of Patients Hospitalized for Acute Myocardial Infarction, By Calendar-Year

	Overall	2006	2007	2008	2009	2010	2011	2012	2013	2014
Number of hospitalizations	1,666,767	210,950	198,832	197,325	184,345	180,279	176,655	178,164	171,762	168,455
**Demographics**										
Age: Mean, SD	79.2 (8.3)	79.2 (8)	79.4 (8.1)	79.5 (8.2)	79.4 (8.2)	79.3 (8.3)	79.3 (8.3)	79.1 (8.4)	78.9 (8.4)	78.7 (8.4)
Male	50.7	48.9	48.9	49.7	50.3	50.6	51.3	51.8	52.8	52.2
**Cardiovascular**										
Percutaneous transluminal coronary angioplasty	12.2	8.1	8.2	8.4	8.5	9.1	15.9	17.2	17.9	18.7
Coronary artery bypass graft surgery	8.9	6.6	6.4	6.4	6.1	6.2	12.0	12.6	12.5	12.3
Congestive heart failure (CC 80)	32.2	32.5	32.8	32.8	32.7	32.3	32.8	32.1	31.2	30.3
Acute Myocardial Infarction (CC 81)	15.7	15.6	15.7	16.0	15.9	16.0	16.4	15.7	15.3	14.5
Unstable angina (CC82)	15.2	15.7	15.5	15.3	15.3	15.0	15.2	15.2	15.1	14.7
Anterior myocardial	9.0	11.3	10.5	9.5	8.9	8.8	8.2	8.0	7.7	7.5

© 2018 Khera R et al. *JAMA Network Open*.

infarction (ICD9 410.00-410.19)										
Other location of myocardial infarction (ICD9 410.20-410.69)	12.7	14.8	14.1	12.7	12.6	12.3	11.9	11.8	11.7	11.5
Chronic atherosclerosis (CC 83 or 84)	80.6	76.5	76.9	77.7	78.0	78.4	84.5	85.1	85.4	85.2
Cardio-respiratory failure and shock (CC 79)	10.1	8.0	9.3	9.7	9.9	10.1	10.9	11.0	11.4	11.6
Valvular or rheumatic heart disease (CC 86)	30.5	31.7	32.0	28.0	27.3	26.8	32.0	32.6	32.1	32.5
**Comorbidity**										
Hypertension (CC 89, 91)	85.5	79.5	82.1	83.7	84.4	85.0	88.9	89.4	89.6	89.6
Stroke (CC 95 or 96)	8.0	8.6	8.4	8.5	8.2	8.0	7.8	7.7	7.4	7.3
Cerebrovascular disease (CC 97 to 99, 103)	20.4	19.1	19.6	20.2	20.2	20.2	21.4	21.5	21.1	20.4
Renal failure (CC 131)	23.9	17.0	20.2	21.5	23.0	24.8	26.7	27.7	28.2	28.4

© 2018 Khera R et al. *JAMA Network Open*.

Chronic obstructive pulmonary disease (CC 108)	30.2	30.8	30.8	29.1	28.5	28.5	31.5	31.2	31.0	30.2
Pneumonia (CC 111 to 113)	24.3	23.7	24.2	25.7	24.9	24.3	24.8	24.0	23.9	22.9
Diabetes and DM complications (CC 15 to 20, 120)	44.7	41.0	41.9	42.8	43.5	44.4	46.9	47.3	47.8	48.0
Protein-calorie malnutrition (CC 21)	5.5	3.6	4.1	4.9	5.4	5.6	6.6	6.8	6.5	6.5
Dementia and senility (CC 49 or 50)	19.2	17.6	18.0	18.6	18.6	18.6	21.1	21.0	20.4	19.9
Hemiplegia, paralysis, functional disability (CC 67 to 69, 100 to 102, 177, 178)	6.2	5.5	5.6	6.1	6.0	6.1	6.7	6.8	6.7	6.7
Vascular or circulatory disease (CC 104, 105)	26.8	24.3	25.2	25.9	26.8	27.0	28.3	28.4	28.2	27.8

© 2018 Khera R et al. *JAMA Network Open*.

Metastatic cancer and acute leukemia (CC 7, 8)	3.9	3.6	3.8	3.9	4.0	3.9	3.9	4.0	3.9	3.9
Trauma (CC 154 to 156, 158 to 162)	30.1	27.8	28.1	28.5	29.6	30.8	31.9	32.0	31.6	31.9
Major psychiatric disorders (CC 54 to 56)	7.4	6.4	6.7	7.0	7.1	7.2	8.3	8.3	8.2	8.2
Liver and biliary disease (CC 25 to 27)	1.2	0.9	1.0	1.0	1.1	1.1	1.4	1.5	1.5	1.7

Values represent %, unless otherwise specified.

© 2018 Khera R et al. *JAMA Network Open*.

eTable 2. Characteristics of Patients Hospitalized for Heart Failure, By Calendar-Year

	**Overall**	**2006**	**2007**	**2008**	**2009**	**2010**	**2011**	**2012**	**2013**	**2014**
Number of hospitalizations	4,054,211	515,324	473,482	464,323	466,125	455,379	438,543	420,849	412,079	408,107
**Demographics**										
Age: Mean, SD	80.9 (8.1)	80.6 (7.9)	80.7 (7.9)	80.8 (8)	80.9 (8.1)	81 (8.1)	81 (8.2)	81 (8.3)	80.9 (8.4)	80.9 (8.4)
Male	44.9	42.4	42.7	44.9	45.0	45.4	45.5	46.1	47.0	45.8
**Cardiovascular**										
Percutaneous transluminal coronary angioplasty	9.7	7.1	7.0	6.8	6.5	6.6	12.7	13.6	14.2	14.8
Coronary artery bypass graft surgery	14.0	11.4	10.6	9.9	9.2	8.8	19.2	19.8	19.7	19.5
Congestive heart failure (CC 80)	78.0	77.5	77.7	77.8	78.2	78.4	78.8	78.5	77.9	77.0
Acute myocardial infarction (CC 81)	10.6	10.4	10.3	10.8	10.8	10.6	10.7	10.8	10.7	10.5
Unstable angina (CC82)	14.4	16.2	15.4	14.9	14.3	13.7	13.6	13.6	13.7	13.6
Chronic atherosclerosis (CC 83, 84)	73.9	73.5	73.4	73.6	72.9	72.5	75.6	75.6	74.6	73.6
Cardio-respiratory	26.2	20.2	22.8	23.4	24.8	26.2	28.1	29.6	30.9	32.4

© 2018 Khera R et al. *JAMA Network Open*.

	Overall	2006	2007	2008	2009	2010	2011	2012	2013	2014
failure and shock (CC 79)										
Valvular or rheumatic heart disease (CC 86)	51.8	51.1	52.2	47.9	47.8	47.7	54.2	55.5	55.4	56.2
**Comorbidity**										
Hypertension (CC 89, 91)	90.8	85.1	87.7	89.4	90.2	90.8	93.5	94.0	94.2	94.4
Stroke (CC 95, 96)	10.2	10.8	10.9	10.7	10.4	10.2	10.0	9.9	9.6	9.4
Renal Failure (CC 131)	47.5	36.8	41.3	43.2	46.0	48.9	51.8	53.7	54.5	54.8
Chronic obstructive pulmonary disease (CC 108)	49.5	49.9	49.8	47.6	47.8	47.9	50.9	51.1	50.8	49.9
Pneumonia (CC 111 to 113)	46.4	43.4	44.8	45.8	46.4	46.6	47.7	47.6	48.3	47.5
Diabetes and DM complications (CC 15 to 20, 120)	53.5	51.3	51.6	52.0	52.6	53.2	55.0	55.5	55.5	55.5
Protein-calorie malnutrition (CC 21)	8.7	5.8	6.3	7.4	8.5	9.1	10.3	10.8	10.8	10.7

© 2018 Khera R et al. *JAMA Network Open*.

	**Overall**	**2006**	**2007**	**2008**	**2009**	**2010**	**2011**	**2012**	**2013**	**2014**
Dementia and Senility (CC 49, 50)	23.0	20.8	21.3	21.7	21.6	21.8	25.1	25.4	25.1	24.6
Hemiplegia, paralysis, functional disability (CC 67 to 69, 100 to 102, 177, 178)	8.0	7.0	7.1	7.6	7.6	7.8	8.7	9.1	9.1	8.8
Vascular or circulatory disease (CC 104, 105)	37.7	34.5	35.3	36.0	37.0	37.4	39.5	40.7	40.3	40.0
Metastatic cancer and acute leukemia (CC 7, 8)	4.3	4.0	4.1	4.2	4.3	4.2	4.3	4.4	4.4	4.5
Trauma (CC 154 to 156, 158 to 162)	38.7	35.6	36.2	36.2	37.5	39.5	40.8	41.3	41.3	41.4
Major psychiatric disorders (CC 54 to 56)	10.1	8.9	9.0	9.4	9.7	9.9	11.0	11.3	11.3	11.3
Liver and biliary disease (CC 25 to 27)	2.7	2.1	2.2	2.2	2.3	2.5	3.0	3.4	3.5	3.7

Values represent %, unless otherwise specified.

© 2018 Khera R et al. *JAMA Network Open*.

eTable 3. Characteristics of Patients Hospitalized for Pneumonia, By Calendar-Year

	Overall	2006	2007	2008	2009	2010	2011	2012	2013	2014
Number of Hospitalizations	3,500,777	470,519	426,232	418,719	383,996	376,956	387,953	365,693	357,377	313,332
**Demographics**										
Age: Mean, SD	80.3 (8.2)	80.2 (8)	80.2 (8.1)	80.5 (8.1)	80.2 (8.2)	80.4 (8.2)	80.4 (8.3)	80.4 (8.3)	80.4 (8.4)	80.3 (8.5)
Male	45.8	44.6	44.7	45.9	46.3	46.6	46.3	46.6	46.6	45.3
**Cardiovascular**										
Percutaneous transluminal coronary angioplasty	5.0	3.4	3.4	3.2	3.3	3.3	6.8	7.3	7.7	8.1
Coronary artery bypass graft surgery	6.4	5.0	4.7	4.5	4.2	4.3	9.0	9.3	9.2	9.3
Congestive heart failure (CC 80)	39.9	39.9	40.0	39.2	39.9	40.1	40.4	40.1	39.6	39.4
Acute myocardial infarction (CC 81)	3.9	3.6	3.6	3.9	3.9	3.9	4.0	4.0	4.1	4.0
Unstable angina (CC82)	6.2	6.9	6.6	6.3	6.3	5.9	6.0	6.0	6.0	6.1
Chronic atherosclerosis (CC 83, 84)	48.5	47.1	47.4	47.4	47.8	47.7	50.2	50.6	49.8	49.5
Cardio-respiratory failure and shock (CC 79)	20.6	15.5	18.3	18.2	19.9	20.9	22.4	23.4	24.3	25.5
**Comorbidity**										
Hypertension (CC	83.6	77.6	79.8	81.5	82.9	83.9	86.6	87.4	87.7	87.8

© 2018 Khera R et al. *JAMA Network Open*.

89, 91)										
Stroke (CC 95, 96)	9.9	10.7	10.5	10.4	10.1	9.8	9.5	9.3	9.0	8.8
Cerebrovascular disease (CC 97 to 99, 103)	21.4	20.3	21.0	21.2	21.5	21.5	22.1	22.2	21.9	21.2
Renal failure (CC 131)	25.6	17.2	20.3	22.3	24.9	26.8	29.1	30.5	31.6	32.5
Chronic obstructive pulmonary disease (CC 108)	56.2	58.3	58.3	54.2	55.2	55.3	56.5	56.4	55.9	55.1
Pneumonia (CC 111 to 113)	45.0	45.3	45.7	45.3	45.7	45.2	45.6	44.7	44.5	43.1
Protein-calorie malnutrition (CC 21)	11.7	8.7	9.2	10.6	11.9	12.4	13.5	13.6	13.6	13.5
Dementia and senility (CC 49, 50)	29.7	28.1	28.4	29.1	28.9	29.0	31.7	31.6	31.2	30.5
Hemiplegia, paralysis, functional disability (CC 67 to 69, 100 to 102, 177, 178)	8.5	7.7	7.7	8.3	8.5	8.5	8.9	9.2	9.1	8.9
Vascular or circulatory disease (CC 104, 105)	30.1	27.0	28.3	28.7	29.9	30.5	31.6	32.2	32.1	31.9

© 2018 Khera R et al. *JAMA Network Open*.

Metastatic cancer and acute leukemia (CC 7, 8)	9.4	8.4	8.8	9.0	9.6	9.7	9.7	10.0	9.9	10.1
Trauma (CC 154 to 156, 158 to 162)	38.8	35.6	36.1	36.5	37.8	39.8	41.0	41.1	41.4	41.9
Major psychiatric disorders (CC 54 to 56)	13.2	11.7	12.0	12.5	12.7	13.0	14.2	14.4	14.6	14.4
Liver and biliary disease (CC 25 to 27)	1.8	1.4	1.5	1.5	1.6	1.6	1.9	2.1	2.2	2.3
Severe hematological disorders (HCC 44)	3.6	2.9	3.6	4.3	4.5	4.6	4.5	2.9	2.3	2.3
Iron deficiency and other/unspecified anemias and blood disease (HCC 47)	53.7	46.9	48.6	50.3	52.2	52.8	59.0	59.5	59.0	58.7
Depression (HCC 58)	20.6	17.0	17.3	17.5	17.9	18.2	24.0	25.2	25.5	25.7
Parkinson's and Huntington's diseases (HCC 73)	4.2	4.3	4.2	4.3	4.2	4.1	4.3	4.1	4.0	4.0
Seizure disorders	5.8	6.0	5.8	5.6	5.5	5.4	5.9	5.9	5.9	5.9

© 2018 Khera R et al. *JAMA Network Open*.

and convulsions (HCC 74)										
Fibrosis of lung and other chronic lung disorders (HCC 109)	16.9	18.0	18.1	17.0	17.5	17.3	17.4	16.1	14.9	14.4
Asthma (HCC 110)	11.5	11.9	11.7	11.1	11.1	11.0	11.6	11.6	11.9	11.9
Vertebral fractures (HCC 157)	5.2	5.2	5.2	5.2	5.2	5.2	5.2	5.2	5.2	5.5

Values represent %, unless otherwise specified.

© 2018 Khera R et al. *JAMA Network Open*.

eTable 4. Interrupted Time Series for Unadjusted In-hospital Mortality, and 30-day Unadjusted Postdischarge Mortality and Readmission Rates

	**Slope (Change in rate per month)**	**Change in slope (at the start of the period)**	**P-value (for change in slope)**
**Acute myocardial infarction**			
**In-hospital mortality**			
Pre-HRRP (Jan 2006 - March 2010)	-0.030 (-0.037, -0.024)		
Post-HRRP announcement (April 2010 - September 2012)	-0.027 (-0.038, -0.016)	0.004 (-0.012, 0.019)	0.64
HRRP penalties (October 2012 - December 2014)	-0.027 (-0.044, -0.010)	0.000 (-0.025, 0.025)	1.00
**30-day post-discharge mortality**			
Pre-HRRP (Jan 2006 - March 2010)	0.004 (-0.003, 0.011)		
Post-HRRP announcement (April 2010 - September 2012)	-0.014 (-0.025, -0.003)	-0.018 (-0.034, -0.002)	0.03
HRRP penalties (October 2012 - December 2014)	-0.017 (-0.035, 0.001)	-0.003 (-0.029, 0.024)	0.84
**30-day post-discharge readmission‡**			
Pre-HRRP (Jan 2006 - March 2010)	-0.004 (-0.012, 0.004)		
Post-HRRP announcement (April 2010 - September 2012)	-0.052 (-0.0644, -0.039)	-0.048 (-0.066, -0.030)	<.001
HRRP penalties (October 2012 - December 2014)	-0.069 (-0.089, -0.049)	-0.017 (-0.046, 0.012)	0.24
**Heart failure**			
**In-hospital mortality**			
Pre-HRRP (Jan 2006 - March 2010)	-0.008 (-0.012, -0.005)		
Post-HRRP announcement (April 2010 - September 2012)	-0.006 (-0.012, 0.000)	0.002 (-0.006, 0.011)	0.58

© 2018 Khera R et al. *JAMA Network Open*.

	Slope (Change in rate per month)	Change in slope (at the start of the period)	P-value (for change in slope)
HRRP penalties (October 2012 - December 2014)	-0.013 (-0.023, -0.003)	-0.007 (-0.022, 0.008)	0.34
**30-day post-discharge mortality**			
Pre-HRRP (Jan 2006 - March 2010)	0.018 (0.012, 0.024)		
Post-HRRP announcement (April 2010 - September 2012)	0.023 (0.014, 0.033)	0.005 (-0.009, 0.018)	0.50
HRRP penalties (October 2012 - December 2014)	0.008 (-0.007, 0.022)	-0.016 (-0.038, 0.007)	0.17
**30-day post-discharge readmission‡**			
Pre-HRRP (Jan 2006 - March 2010)	0.013 (0.007, 0.019)		
Post-HRRP announcement (April 2010 - September 2012)	-0.051 (-0.061, -0.040)	-0.063 (-0.078, -0.049)	<.001
HRRP penalties (October 2012 - December 2014)	-0.040 (-0.057, -0.024)	0.010 (-0.014, 0.034)	0.40
**Pneumonia**			
**In-hospital mortality**			
Pre-HRRP (Jan 2006 - March 2010)	-0.001 (-0.005, 0.003)		
Post-HRRP announcement (April 2010 - September 2012)	-0.024 (-0.031, 0.017)	-0.024 (-0.031, -0.017)	<.001†
HRRP penalties (October 2012 - December 2014)	-0.025 (-0.037, -0.013)	-0.025 (-0.037, -0.013)	0.90
**30-day post-discharge mortality**			
Pre-HRRP (Jan 2006 - March 2010)	0.021 (0.015, 0.026)		
Post-HRRP announcement (April 2010 - September 2012)	0.003 (-0.006, 0.012)	-0.018 (-0.030, -0.005)	0.007†
HRRP penalties (October 2012 - December 2014)	0.000 (-0.015, 0.014)	-0.003 (-0.024, 0.018)	0.77

© 2018 Khera R et al. *JAMA Network Open*.

	**Slope (Change in rate per month)**	**Change in slope (at the start of the period)**	**P-value (for change in slope)**
**30-day post-discharge readmission‡**			
Pre-HRRP (Jan 2006 - March 2010)	0.015 (0.007, 0.023)		
Post-HRRP announcement (April 2010 - September 2012)	-0.036 (-0.049, -0.022)	-0.050 (-0.070, -0.031)	<0.001
HRRP penalties (October 2012 - December 2014)	-0.023 (-0.045, 0.000)	0.013 (-0.019, 0.045)	0.42

*Slopes represent the slope of the regression line in the corresponding period, representing the average change in mortality and readmission rates (%) over a month in this period.
†Significant, after applying Holm-Bonferroni adjustment to maintain a family-wide type I error rate of 0.05. (All P-values for the outcomes of in-hospital and 30-day post-discharge mortality that are not marked † are not significant.)
‡P-value threshold for significance for the secondary outcome of 30-day post-discharge readmission was 0.05.

© 2018 Khera R et al. *JAMA Network Open*.

eTable 5. Interrupted Time Series for Risk-Adjusted In-hospital/30-day Postdischarge Mortality and 30-day Post-admission Mortality

	**Slope (Change in rate per month)**	**Change in slope (at the start of the period)**	**P-value (for change in slope)**
**Acute myocardial infarction**			
**In-hospital or 30-day post-discharge mortality**			
Pre-HRRP (Jan 2006 - March 2010)	-0.024 (-0.034, -0.015)		
Post-HRRP announcement (April 2010 - September 2012)	-0.002 (-0.017, 0.014)	0.023 (0.000, 0.045)	0.05
HRRP penalties (October 2012 - December 2014)	-0.016 (-0.040, 0.009)	-0.014 (-0.050, 0.023)	0.45
**30-day post-admission mortality**			
Pre-HRRP (Jan 2006 - March 2010)	-0.020 (-0.029, -0.011)		
Post-HRRP announcement (April 2010 - September 2012)	0.000 (-0.014, 0.014)	0.021 (0.000, 0.041)	0.05
HRRP penalties (October 2012 - December 2014)	-0.015 (-0.037, -0.008)	-0.015 (-0.048, 0.018)	0.37
**Heart failure**			
**In-hospital or 30-day post-discharge mortality**			
Pre-HRRP (Jan 2006 - March 2010)	-0.013 (-0.028, 0.001)		
Post-HRRP announcement (April 2010 - September 2012)	0.014 (-0.009, 0.038)	0.028 (-0.007, 0.062)	0.11
HRRP penalties (October 2012 - December 2014)	-0.017 (-0.055, 0.021)	-0.031 (-0.087, 0.024)	0.27
**30-day post-admission mortality**			
Pre-HRRP (Jan 2006 - March 2010)	-0.009 (-0.022, 0.003)		
Post-HRRP announcement (April 2010 - September 2012)	0.013 (-0.008, 0.034)	0.022 (-0.008, 0.052)	0.16
HRRP penalties (October 2012 - December 2014)	-0.013 (-0.046, 0.021)	-0.025 (-0.075, 0.024)	0.31
**Pneumonia**			
**In-hospital or 30-day post-discharge mortality**			
Pre-HRRP (Jan 2006 - March 2010)	-0.009 (-0.017, -0.001)		

© 2018 Khera R et al. *JAMA Network Open*.

	Slope (Change in rate per month)	Change in slope (at the start of the period)	P-value (for change in slope)
Post-HRRP announcement (April 2010 - September 2012)	-0.015 (-0.028, -0.001)	-0.006 (-0.025, 0.014)	0.58
HRRP penalties (October 2012 - December 2014)	-0.017 (-0.040, 0.006)	-0.002 (-0.035, 0.030)	0.89
**30-day post-admission mortality**			
Pre-HRRP (Jan 2006 - March 2010)	-0.006 (-0.013, 0.001)		
Post-HRRP announcement (April 2010 - September 2012)	-0.010 (-0.022, 0.002)	-0.004 (-0.021, 0.013)	0.65
HRRP penalties (October 2012 - December 2014)	-0.016 (-0.036, 0.004)	-0.006 (-0.034, 0.023)	0.69

*Slopes represent the slope of the regression line in the corresponding period, representing the average change in mortality and readmission rates (%) over a month in this period.

© 2018 Khera R et al. *JAMA Network Open*.
